# Damaraland mole-rats do not rely on helpers for reproduction or survival

**DOI:** 10.1093/evlett/qrad023

**Published:** 2023-05-29

**Authors:** Jack Thorley, Hanna M Bensch, Kyle Finn, Tim Clutton-Brock, Markus Zöttl

**Affiliations:** Department of Zoology, University of Cambridge, Cambridge, United Kingdom; Kalahari Research Centre, Kuruman River Reserve, Van Zylsrus, South Africa; Kalahari Research Centre, Kuruman River Reserve, Van Zylsrus, South Africa; Department of Biology and Environmental Science, Linnaeus University, Kalmar, Sweden; Kalahari Research Centre, Kuruman River Reserve, Van Zylsrus, South Africa; Department of Zoology and Entomology, Mammal Research Institute, University of Pretoria, Pretoria, South Africa; Department of Zoology, University of Cambridge, Cambridge, United Kingdom; Kalahari Research Centre, Kuruman River Reserve, Van Zylsrus, South Africa; Department of Zoology and Entomology, Mammal Research Institute, University of Pretoria, Pretoria, South Africa; Kalahari Research Centre, Kuruman River Reserve, Van Zylsrus, South Africa; Department of Biology and Environmental Science, Linnaeus University, Kalmar, Sweden

**Keywords:** sociality, cooperative breeding, helper effects, philopatry, family living

## Abstract

In eusocial invertebrates and obligate cooperative breeders, successful reproduction is dependent on assistance from non-breeding group members. Although naked (*Heterocephalus glaber*) and Damaraland mole-rats (*Fukomys damarensis*) are often described as eusocial and their groups are suggested to resemble those of eusocial insects more closely than groups of any other vertebrate, the extent to which breeding individuals benefit from the assistance of non-breeding group members is unclear. Here we show that, in wild Damaraland mole-rats, prospective female breeders usually disperse and settle alone in new burrow systems where they show high survival rates and remain in good body condition—often for several years—before being joined by males. In contrast to many obligate cooperative vertebrates, pairs reproduced successfully without non-breeding helpers, and the breeding success of experimentally formed pairs was similar to that of larger, established groups. Though larger breeding groups recruited slightly more pups than smaller groups, adult survival was independent of group size and group size had mixed effects on the growth of non-breeders. Our results suggest that Damaraland mole-rats do not need groups to survive and that cooperative breeding in the species is not obligate as pairs can—and frequently do—reproduce without the assistance of helpers. While re-emphasizing the importance of ecological constraints on dispersal in social mole-rats, the mixed effects of group size in our study suggest that indirect benefits accrued through cooperative behavior may have played a less prominent role in the evolution of mole-rat group-living than previously thought.

## Introduction

Group living animals show large variation in reproductive skew, ranging from communal breeders where all females breed, to some insect societies where group size can reach millions and reproduction is limited to a small number of individuals that are irreversibly specialized as breeders (Boomsma & Gawne, 2018; Clutton-Brock, 2016; Crespi & Yanega, 1995; Oster & Wilson, 1978; Sherman et al., 1995). Group living species also show large variation in the expression of alloparental care. In the obligately eusocial insect societies, queens specialize on egg laying and workers provide all necessary brood care, whereas in the smaller groups of primitively eusocial insects and cooperatively breeding vertebrates, the importance of non-breeding helpers varies. In some, non-breeders generate relatively weak benefits to breeders and helping can be considered mostly facultative, while in others helping is obligate and successful reproduction typically depends on the presence of non-breeding helpers who play a key role in offspring development (Cant, 2012; Cockburn, 1998; Field et al., 2006; Koenig & Dickinson, 2016; Solomon & French, 1997). Finally, there are some family-living species where retained offspring provide no direct assistance to breeding individuals and instead participate in mutually beneficial activities such as territory defense (Downing et al., 2020; Ekman & Griesser, 2016; Griesser et al., 2017). Clarifying the role of non-breeders across reproductively skewed societies is crucial for identifying the pathways to advanced forms of sociality across the tree of life, and for understanding the ways in which life histories can impede or facilitate social evolution (Boomsma & Gawne, 2018; Chak et al., 2017; Cooper & West, 2018; Emlen, 1982, 1995; Griesser et al., 2017; Lukas & Clutton-Brock, 2012).

While reproductive skew and alloparental care are often closely associated, this is not always the case. In the social African mole-rats (*Bathyergidae*), for example, groups are typically nuclear families with high reproductive skew (being limited to a single breeding female in each group: Bishop et al., 2004; Braude, 2000; Burland et al., 2004; Caspar et al., 2021; Faulkes & Bennett, 2021; Jarvis & Bennett, 1993; Patzenhauerová et al., 2013; Spinks et al., 2000) but little alloparental care. Non-breeders of both sexes will groom and retrieve pups born to the breeding female if they stray from the nest (Mooney et al., 2015; Watarai et al., 2018; Zöttl et al., 2016, 2018), and may help to thermoregulate pups at early stages of development (Kotze et al., 2008; Vavrušková et al., 2022). However, the principal cooperative activities of non-breeders involve the maintenance of a network of tunnels that provide access to underground plant tubers, and the transportation of tubers to food stores that are available to all group members (Bennett & Faulkes, 2000; Brett, 1991a; Zöttl et al., 2016). As the tubers on which mole-rats rely are often patchily distributed, increases in group size have been associated with increases in the probability of finding food, and cooperative foraging is often assumed to bring general benefits to all group members. The idea that there are strong benefits of group-living is therefore implicit in most discussions of mole-rat sociality (Bennett, 1990; Burda et al., 2000; Jarvis et al., 1994, 1998; Lovegrove, 1991; Young et al., 2015), and the presence of a non-breeder “workforce” has been suggested to be particularly important in arid environments where the energetic constraints on burrowing are high (Faulkes et al., 1997; Jarvis et al., 1998). By implication, it has also been suggested that kin selection has played a major role in the evolution of mole-rat sociality, with individuals acquiring inclusive fitness benefits by remaining in their natal group and helping (Bennett & Faulkes, 2000; Faulkes & Bennett, 2021; Jarvis et al., 1994).

As longitudinal studies of wild mole-rat populations are uncommon (Braude, 2000; Brett, 1991b; Faulkes & Bennett, 2021; Torrents-Ticó et al., 2018), direct evidence that non-breeders confer reproductive and survival benefits to breeders under natural conditions is rare. The only study to have explored the effect of group size on fitness-related traits in wild mole-rats to date comes from a population of Damaraland mole-rats in Namibia, where it was found that larger groups recruited more offspring (Young et al., 2015). In the same study, juvenile survival was independent of group size and juvenile growth rate was reduced in large groups, possibly through competition. Breeding Damaraland mole-rat females have also been shown to have lower workloads than non-breeders in a separate wild population (Francioli et al., 2020) and in captive animals (Houslay et al., 2020), alluding to possible helper effects in both settings. However, with the cooperative actions of non-breeders being largely indirect, it cannot be assumed that breeders profit from having non-breeding group members present. Group living has been lost at least once in the African mole-rats following the divergence of the clade from a common social ancestor (Faulkes & Bennett, 2021), and it is possible that non-breeders bring relatively limited fitness benefits to breeders in social mole-rat populations; and that extended philopatry has been selected for because it allows individuals to optimize the timing of their dispersal and maximize their own lifetime reproductive success. Ultimately, it remains unclear whether breeding pairs without non-breeding helpers are less successful in raising young to independence, and it is still untested whether individuals in small groups experience higher mortality.

In this study we investigate how new breeding groups emerge and determine whether reproductive success depends on the presence of non-breeders in a wild population of Damaraland mole-rats living in the southern Kalahari Desert (Supplementary Figure S1). We first describe how new groups form and then we assess whether individuals living in groups (breeders and non-breeders) experience higher survivorship than individuals that have dispersed and settled solitarily. By experimentally creating breeding pairs in the field, we also examine whether the reproductive output of breeders lacking non-breeding group members is lower than that of breeders in established groups which have access to a potential workforce. Lastly, we use demographic information collected across 7 years of field study to analyze the associations of group size with (a) reproductive success, (b) adult survival, and (c) growth of recruited offspring, testing the prediction that large group size is associated with increases in reproductive success and survival, and enhances growth among young individuals.

## Methods

### General methods

We captured Damaraland mole-rats in the area surrounding the Kuruman River Reserve (−26.978560, 21.832459) in the Kalahari Desert of South Africa between September 2013 and May 2020 (Supplementary Figure S1). Field work was carried out at two neighboring localities, the Kuruman River Reserve (“Kuruman”), and the Lonely farm (“Lonely,” Supplementary Figures S2 and S3). The two locations form a continuous population but because of the time intensive nature of mole-rat trapping they tended to be sampled in discrete periods (Supplementary Figures S4 and S5, Supplementary Table S1), hereafter referred to as “trapping windows.” For logistical reasons, trapping stopped at Lonely in July 2017.

The study site is characterized by cold and dry winters (May–September) and very hot summers (October–April), though large fluctuations in air temperature occur throughout the year. The region suffers episodic droughts, but when rain does fall, it is usually concentrated into short summer downpours (Supplementary Figure S1). The climatic conditions and soil characteristics of our study site are similar to those of other locations where medium to long-term studies of Damaraland mole-rats have been conducted over the past four decades (Bennett & Faulkes, 2000; Mynhardt et al., 2021; Torrents-Ticó et al., 2018) and are representative of the species distribution generally (Supplementary Figures S6-S8). Some basic demographic comparisons of the different populations are provided in Supplementary Table S2.

**Figure 1.**
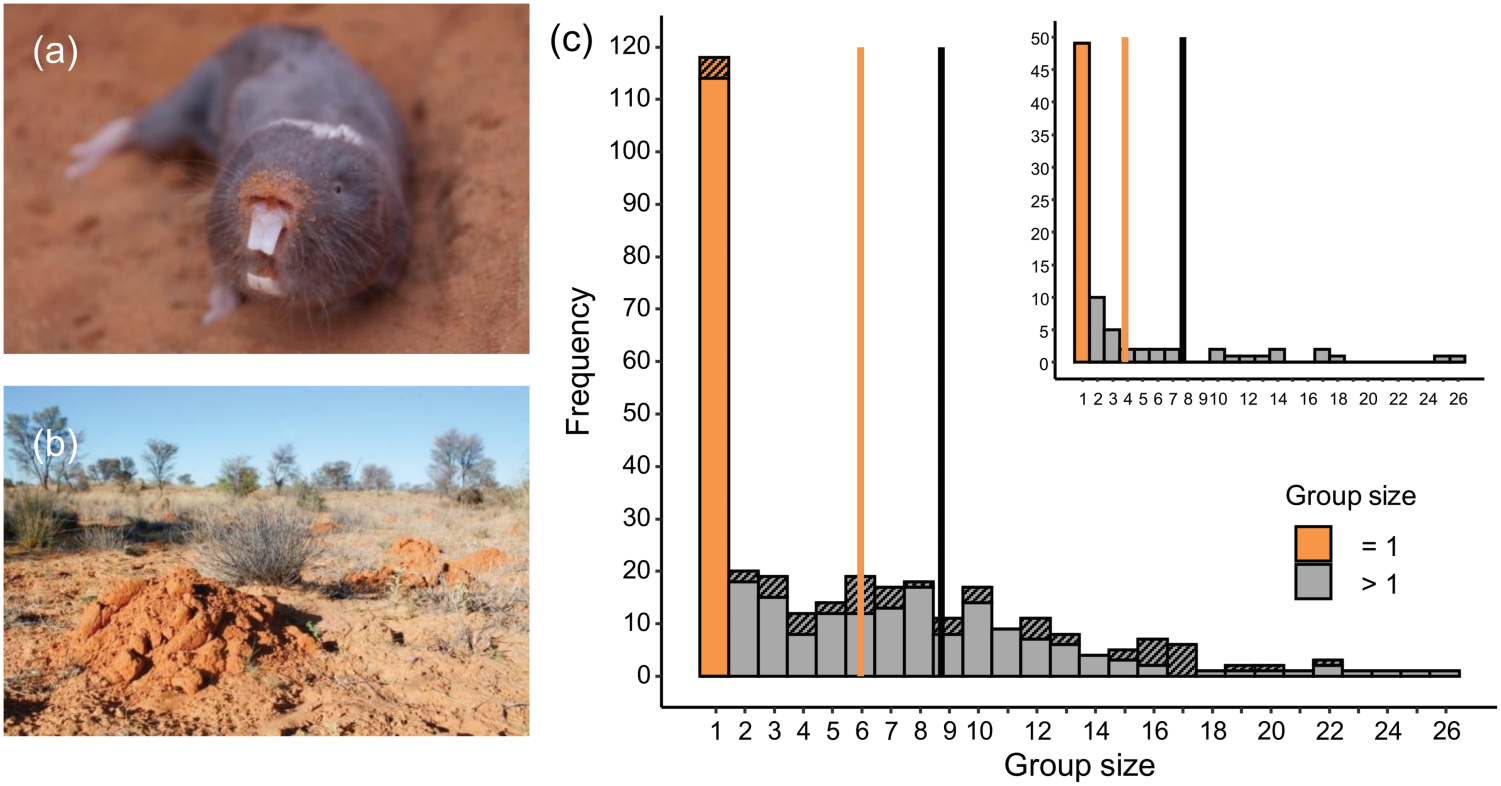
Damaraland mole-rat group sizes in the southern Kalahari. (A) An adult Damaraland mole-rat. (B) A line of sand mounds that is created by a group of mole-rats when they dig foraging tunnels underground. (C) The distribution of group sizes in the study population at the Kuruman River Reserve. The main histogram displays group size frequencies across all captures, while the inset histogram displays the group size at first capture for each unique group (*n* = 84, including single individuals). A large proportion of the population were captured as single individuals (orange), who were almost exclusively females. The vertical lines display the mean group size either including (orange, lower value) or excluding (black, upper value) these single individuals, and for all captures, we distinguish between complete and incomplete group captures by hatching the latter (mean for all group captures = 5.91, 1 *SD* = 5.61; mean for all captures of groups > 1 = 8.67, 1 *SD* = 5.30). Removing the 15.2% of incomplete captures had limited influence on the mean group size (not displayed, mean for all complete group captures = 5.45, 1 *SD* = 5.51; mean for all complete captures of groups > 1 = 8.54, 1 *SD* = 5.32).

Groups of mole-rats in the wild are revealed by the lines of mounds that they extrude when excavating their tunnel systems (Figure 1). We captured groups periodically at 6 to 12-month intervals using modified Hickman traps that were baited with sweet potato and were positioned into tunnel systems once accessed by digging. The traps were checked every 2–3 hrs throughout the day and night. On capture, animals were placed into a closed box and provided with food and shelter. Intermittently, we transported individuals back to the laboratory where they were sexed, weighed to the nearest gram, and measured for various morphometrics. The breeding females were commonly the largest female in their group and could be easily identified from their perforated vagina and prominent teats. All captured individuals were marked with passive integrated transponder (PIT) tags on first capture to allow for individual recognition on recapture. After sampling, groups were housed temporarily in semi-natural tunnel systems in the laboratory and provided with food, nesting material, and sand. The complete group was assumed to have been captured after an absence of activity at the trap sites for 24 hrs, after which point the animals were returned to their tunnel system. The average time to capture a group was 2.73 ± 1.67 days (mean ± *SD*).

Between September 2013 and May 2020, we captured 752 unique individuals (368 females, 384 males) at the two sites (*n* = 1,941 individual captures). The mean recapture rate of individuals across successive trapping windows was 73.1 ± 10.8% at Kuruman and 54.8 ± 14.4% at Lonely (mean ± *SD*; Supplementary Table S1). In total, analyses used data from 328 group captures that were carried out at 84 groups with an average of 3.90 ± 2.99 captures per group (mean ± *SD*; Supplementary Figure S3). We defined groups as individuals repeatedly found at the same trapping location, and in most cases the same breeding female remained at a group for their duration. Provided that most individuals captured within the same area were known to originate from the same group, we therefore continued to define the group as such. On several occasions a new group had moved into the tunnel system previously occupied by another group. The initial group were then either found elsewhere or never recaptured and likely extinct. Single individuals—either new unknown individuals or known individuals that had dispersed from an established group—were assigned their own unique group ID, which was retained if they were later joined by an immigrant partner and started breeding in the same area.

In 15.2% of group captures, continued activity at trapping sites indicated that the complete group had not been captured by the end of the week of trapping. The placement of an RFID panel antenna above active foraging tunnels suggested that when this was the case it was usually a single, large nonreproductive individual evading capture (see Francioli et al., 2020 for methodology). The decision to include or exclude these incomplete captures depended on the analysis: group-level analyses excluded incomplete captures whereas individual-level analyses included them (a summary of the data and models used for the various analyses is provided in Supplementary Table S10).

### State-related survivorship among females

To estimate the survivorship of females in different states we fitted a multi-state Markov model (MSM). Such models are typically used in a medical setting for “panel data,” where a continuously observed process—like disease progression—is measured only at discrete time points—when people choose to visit the hospital. The timing of transitions between states can then be estimated indirectly under the assumption that the next state in a sequence of states depends only on the current state, and not on the history of transitions or on the time spent in the prior state (Jackson, 2011). Our longitudinal data bears a panel-like structure, with individuals and groups being periodically captured (or not) in different life history stages or “states.” The model then estimates the probability that individual mole-rats transition between different states. Here, we present information on survival probability from each state.

Information on individual capture histories was used to assign females to one of four states relative to the time since their first capture: (*i*) *non-breeder* in their natal group, (*ii*) *single female*, (*iii*) *breeding female* out of their natal group, (*iv*) *disappeared*/*dead* (Supplementary Figure S10). For model fitting purposes, in the small number of cases where females inherited a breeding position within their natal group (*n* = 7), these females were categorized in the same state as females that acquired a breeding position out of their natal group (*iii*). To define state *iv* (disappeared/died), we incorporated information from the trapping windows at Kuruman and Lonely. If an individual had not been recaptured for at least two consecutive trapping windows, we assumed that it had dispersed or died at some point between its last live capture and the start of the subsequent trapping window and was given an extra row of data reflecting this; the model was then parameterized so that the timing of disappearance or death was not exact (Jackson, 2011). Similarly, if individuals were captured within the last trapping window at each location, we assumed that they were still alive at the end of that trapping window (i.e., the end of the study). They were then given an extra row of data reflecting this assumption and were censored at this point, which in the most extreme case assumed that individuals remained in their last captured state for a further 80 days (Supplementary Figure S10). The multi-state analysis included 326 females that were captured as a *non-breeder* in their natal group at some point in the study, 45 females captured as a *single female*, and 46 captured as a *breeding female*. To explore whether group size affected the likelihood that non-breeding females or breeding females died/disappeared, we fitted an additional model that included a group size covariate for each of these transitions. The multi-state models were fitted in the *msm* package (Jackson, 2011; Supplementary Figure S9 for multi-state diagram). When reporting hazard ratios and relative likelihoods from the multi-state models we considered cases where the 95% confidence intervals did not overlap one as indicating a biologically important effect, while also emphasizing effect sizes. To directly compare survivorship among the different classes of female, we calculated the ratio of the transition intensities from each alive state (*i*, *ii*, or *iii*) to death/disappearance (*iv*), with 95% confidence intervals computed using the delta method (*qratio.msm* function).

### Within-group recruitment

We used two approaches to investigate the role of group size on within-group recruitment. In the first, we analyzed recruitment longitudinally across the duration of the study. In the second, we experimentally created new groups in the wild through the introduction of unfamiliar adult males to solitary females. The recruitment rate of newly created pairs was then compared to that of established groups that were captured and recaptured within the same time period. The benefit of the second approach is that it allows for a direct test of whether groups in the early stages of group formation were less productive than established groups. Any new individuals caught at a group within one year that were lighter than 100 g (males) or 80 g (females) were assumed to be recruited from within the group, whereas any new individuals heavier than these cut-offs were assumed to represent out-of-group immigrants. Two pieces of information support this decision. Firstly, body mass growth curves demonstrate that individuals under these thresholds are very unlikely to be older than 1 year of age at the time of capture (see below; and Thorley, 2018 for a comparison in captivity). Secondly, we have no evidence to suggest that individuals disperse in their first year of life—across hundreds of capture–recapture events we have never recaptured an individual in a new group shortly after their first capture as a juvenile (<50 g). The longitudinal analysis used information from every capture–recapture event where a group was captured and then recaptured within a 100 to 365-day period; where a resident breeding female was present; and where at least one large male was retained across the two captures. The analysis is therefore restricted to active breeding groups. In total, this produced a dataset of 78 group-level capture–recapture events that took place in 33 groups with a mean trapping interval of 214.2 ± 52.1 days (mean ± *SD*). The number of recruits was modeled using a generalized linear mixed effects model (GLMM) with Poisson error in the *glmmTMB* package (Brooks et al., 2017). We included fixed effects of group size at first capture, the body mass of the breeding female at the capture, and rainfall. We also included a single random effect of group identity, and the logged time interval between capture and recapture as an offset term. Rainfall was calculated as the geometric mean monthly rainfall in the year preceding the first capture as this provided the best fit to the data (lowest AIC score) when compared to models fitted to the total or arithmetic mean rainfall. After standardizing relative to the trapping duration, the mean 6-monthly recruitment to groups was 2.12 ± 1.82 individuals (mean ± *SD*). Continuous variables were *z*-score transformed prior to model fitting and showed no sign of collinearity (variance inflation factors < 1.04).

For the experimental approach, we captured eight solitary females between April 16, 2016 and May 29, 2016. Solitary females were brought back into the laboratory and were paired with wild males captured concurrently from intact breeding groups. When pairing, we first isolated males and females in their own temporary tunnel system and exposed them to the odor of their prospective partner for 24 hr. After this period, we introduced the male to the female for 24 hr in her laboratory tunnel system, before returning both individuals together to the female’s tunnel system in the wild. We used large adult males originating from groups >3 km from the focal solitary female for all pairings, so it is highly unlikely that pairings were conducted between close relatives and/or familiar individuals. To compare the recruitment rate of new pairs to that of established groups captured over the same period, we used a Welch’s *t*-test (again standardizing recruitment rate to a 6 monthly measure). Groups where a male was removed were not included in the established group category in case that male was a breeder in his original group, which would necessarily reduce the rate of recruitment. The mean trapping interval did not differ between new pairs (249.89 ± 94.34 days; mean ± *SD*) and established groups (306.88 ± 64.39 days; mean ± *SD*; Welch’s *t*-test: *t* = −1.47, df = 14.14, *p* = .16).

### Early-life growth

As the age of wild-caught individuals was unknown, we modeled growth using interval equations that estimated the change in body mass and upper incisor width of individuals across successive capture events. The upper incisors are a reliable measure of skeletal size in Damaraland mole-rats and are the main apparatus of digging (Young & Bennett, 2010). Incisor width was measured at the widest point using digital calipers (to the nearest 0.01 mm). All measurements were taken in duplicate by two observers, and we used the mean of these two measurements. Previous studies show that the shape of growth in captive mole-rats is concave and can be approximated by a monomolecular curve (Thorley, 2018). To allow for a similar shape of growth in the wild, we parameterized the interval equation as a von Bertalanffy growth curve (Thorley & Clutton-Brock, 2019) and fitted the curve as a nonlinear mixed effects model (NLMM) in the *nlme* package (Pinheiro et al., 2022). Body mass or incisor width of an individual at each recapture was then estimated as:


$$\begin{array}{l} S2_{i}=A-\left(A-S1_{i}\right)e^{-k.D_{i}}+\varepsilon_{i} \end{array}$$

where S1_*i*_ and S2_*i*_ are the size at capture and recapture, respectively, for individual *i*. A is the estimated population-level asymptotic size, *k* is the growth rate constant, *D*_*i*_ is the time difference between capture and recapture. ε_i_ is the normally distributed error with mean 0 and variance *σ*^2^.

We fitted separate models to males and females for each size metric, using all information from individuals that were recaptured at least once, and where the recapture interval fell between 90 and 365 days. The female body mass dataset comprised 381 repeat capture events (*n* = 193 females, mean/female = 1.96 ± 1.21; mean ± *SD*) and excluded any weight data from females once they were known to be a breeder, thus removing any effect of status or pregnancy. The male body mass dataset consisted of *n* = 456 repeat capture events (*n* = 214 males, mean/male = 2.13 ± 1.54; mean ± *SD*). The female and male incisor width datasets consisted of 328 and 381 repeat capture events respectively (*n* = 193 females, mean/female = 1.82 ± 1.16; *n* = 198 males, mean/male = 1.92 ± 1.33; mean ± *SD*). In each model we specified a random effect of individual identity at the level of the asymptote and the growth rate constant, and to aid convergence, random effects were modeled as uncorrelated. Likelihood ratio tests indicated that the random effects for group identity accounted for negligible amounts of variance and were therefore not included in the final growth models.

We then extended the initial growth model by incorporating a standardized group size term:


$$\begin{array}{l} S2_{i}=\left(A+A_{GS}.GS\right)-\left(\left(A+A_{GS}.GS\right)-S1_{i}\right)e^{-\left(k+k_{GS}.GS\right).D_{i}}+\varepsilon_{i} \end{array}$$

Here, *A*_GS_ and *k*_GS_ estimate the change in asymptotic mass and growth rate constant with changing group size, *GS*. Group size was taken as the group size on initial capture, which was highly correlated with recapture group size (*r* = 0.78, df = 835, *p* < .001). The results were qualitatively unaffected by the underestimation of group size for incomplete captures, being unchanged when adding one to group size for all incomplete captures (under the assumption that a single individual evaded capture). Group size was *z*-score transformed prior to model fitting, such that *A* and *k* are estimated at the mean group size experienced by each sex.

Analyses were conducted in R version 4.2.1 (R Core Team, 2022), and all code and data are provided online (https://github.com/JThor1990/DMR_GroupSizeEffects). Summary statistics of the raw data are reported as the mean ± 1*SD*; model estimates are reported as either the mean ± 1SEM, or as a hazard ratio ± 95% confidence intervals.

## Results

### Population structure and distribution of group sizes

The mean group size across all captures at our study site between 2013 and 2020 was 5.91 ± 5.61 individuals (mean ± *SD*; max = 26; *n* = 328 group captures; Figure 1). Of the 84 unique groups that were captured, 49 were of a single individual on the first capture, 94% of which were females (*n* = 46). Excluding this large number of single individuals, the mean group size was 8.67 ± 5.30 individuals (mean ± *SD*; *n* = 210 group captures), which can be taken to represent the average size of a breeding group in the population. The distribution of group sizes is comparable to other populations of Damaraland mole-rats (Supplementary Table S2). Across the duration of the study only one reproductively active “breeding” female was present at a group at any given time.

### Dispersal and routes to breeding

Males and females dispersed from their natal group at a similar age (two-state Markov model; hazard ratio of males relative to females = 1.125, 95% CI = [0.864,1.476]; see SI for philopatry analyses, Supplementary Figure S11), approximately 1.80 years after their first capture for males (95% CI [1.50,2.15]), and 2.02 years after their first capture for females (95% CI [1.66,2.47]).

The large number of single females reflects a divergence in the life history trajectories of the two sexes after departure from their natal group. Post-dispersal, females usually settled as single individuals, whereas males left their natal group and sought to locate females and seldom settled on their own. We identified 23 females in our population whose route to breeding could be determined accurately. Of these 23, 60.9% (*n* = 14) were females that had dispersed from their group, settled singly, and were later joined by one or more emigrant males to start a nascent breeding group. Two further females became breeders through territory budding, having dispersed and subsisted alone in a burrow system directly adjacent to but separate from their natal burrow, with males subsequently immigrating in. Lastly, 30.3% of the breeding females (*n* = 7) inherited a breeding position in their natal burrow system after the disappearance of the previous breeding female. We never observed any females that immigrated into established groups and cases of female inheritance were always associated with the loss of the incumbent breeding female: either because group collapse left a single female that was later joined by a dispersing male forming a new pair (*n* = 3), or because a natal female directly replaced the breeding female within a larger group (*n* = 4).

For males, joining single females (*n* = 16, 55.2%) or immigrating into established groups (*n* = 13, 44.8%) were both major routes through which males accessed unfamiliar females. Without paternity information it is unknown whether these males were the father of all offspring born post-immigration. Cases of multiple male immigration (from either the same or from different groups) were observed but were infrequent (*n* = 3 cases). We found no evidence that males inherited breeding positions in their natal group.

### State-related survivorship among females

**Figure 2.**
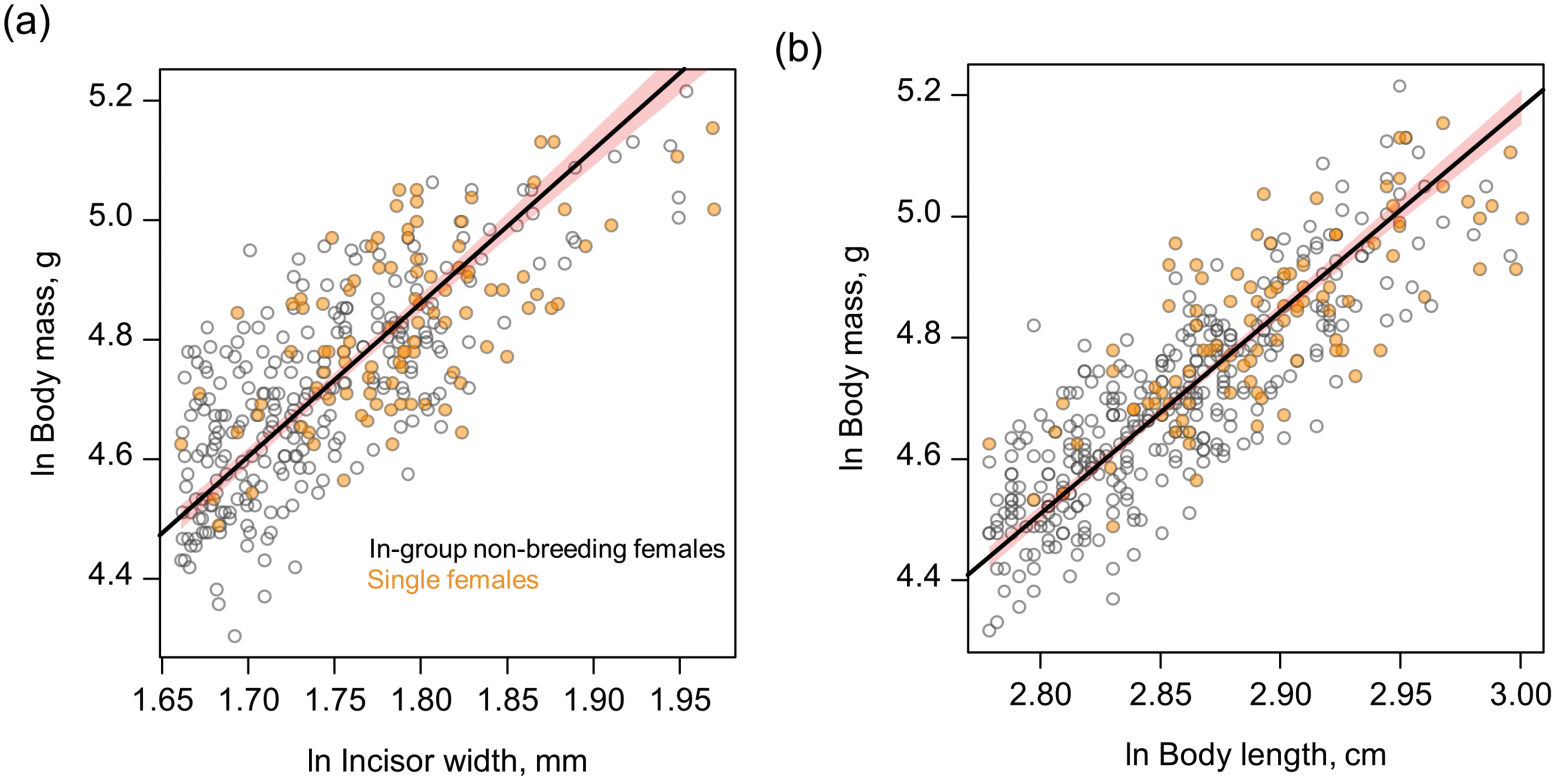
The body condition of dispersed single females compared to size-matched in-group non-breeding females. Plots display the scaled major axis regression (SMA) between incisor width and body mass (A) and between body length and body mass (B). Points display the raw data and lines display the predicted regression through all the data, with 95% bootstrapped confidence intervals given by the shaded area. Allowing the two classes of females to have different intercepts and slopes did not improve model fit: the two classes of females did not differ in mean body condition, or in the change in body condition with increasing skeletal size. Incisor width data (*n* = 353) includes 255 measures from in-group non-breeding females (*n* = 151 unique individuals) and 98 from single females (*n* = 44 unique individuals). Body length data (*n* = 419) includes 320 measures from in-group non-breeding females (*n* = 187 unique individuals) and 99 from single females (*n* = 44 unique individuals).

**Figure 3.**
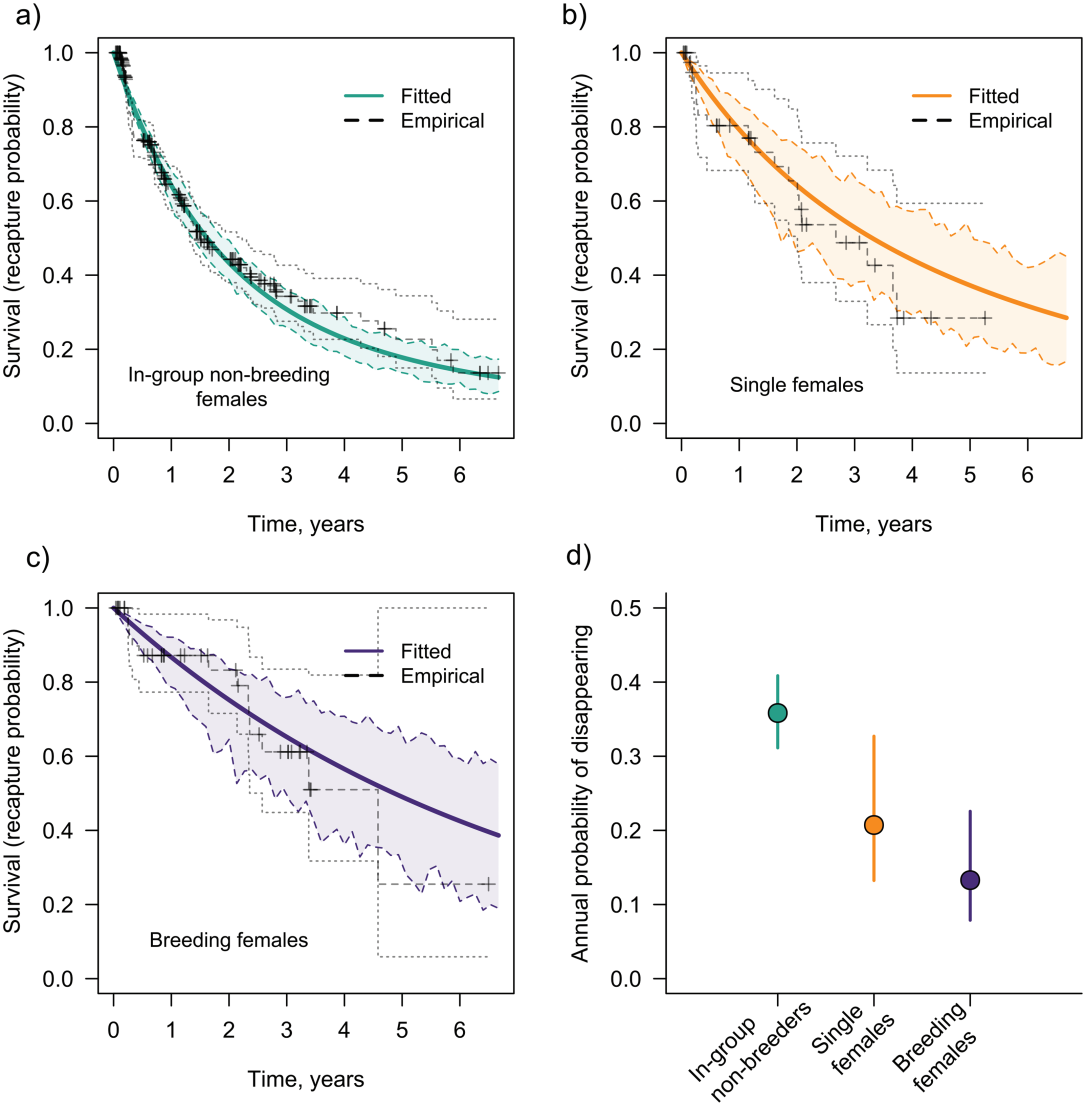
State-related survivorship of female Damaraland mole-rats: in-group non-breeders (A), single females (B), and breeding females (C). Solid lines display the expected probability of survival from each state as estimated from the multi-state Markov model (see Methods). Dotted lines display the empirical Kaplan–Meier estimate of survival probability, with crosses denoting cases of right-censoring. Here, survival strictly refers to disappearance from the study population, and the predicted annual probability of disappearance (mean ± 95% CI) is given in (D). “Disappearance” therefore combines cases where individuals died within their group (in-situ mortality) and cases where mortality occurred during dispersal (when individuals left their current group and state and were not recaptured thereafter). For in-group non-breeders, mortality during dispersal is likely to account for a large proportion of disappearances (Supplementary Table S5) as increases in body mass and rainfall both increased the probability of disappearance (see Text). The multi-state model combined information on 326 *in-group non-breeding females*, 45 single females, and 46 breeding females (though females could appear in multiple states). Note that one female had been recaptured as a single individual for 5.26 years by the endpoint of the study and was right censored at that point.

Single females maintained good body condition over extended periods, frequently over several years, and their body condition was equivalent to that of non-breeding females of similar skeletal size (Figure 2, body condition analyses outlined in Supplementary Material). Single females disappeared from the population at rates intermediate between those of breeders and those of non-breeders (Figure 3 and Supplementary Table S4): their estimated annual survival (79.3%) was higher than that of in-group non-breeding females (64.2%) and was lower than that of breeding females (86.7%). Overall, in-group non-breeding females were 1.91 times more likely to disappear from the population than single females (MSM: ratio of transition probabilities; 95% CI [1.07,3.43]), and 3.28 times more likely to disappear than breeding females (MSM: 95% CI [1.84,5.82]). The rate of disappearance of single females was higher than that of breeding females, with the former being 1.71 times more likely to disappear than the latter, though this effect was not significant (MSM: 95% CI [0.78,3.77]; Figure 3C).

The disappearance of non-breeders from groups was often likely to be due to mortality that occurred during dispersal rather than in-situ mortality as increases in weight and rainfall both increased the probability of disappearance (Supplementary Table S5): as would be expected if dispersal increases when individuals are in better condition (or older) and when environmental conditions are more favorable. The low incidence of recaptures outside the natal group and the low incidence with which unknown adults immigrated into the study population suggests that dispersal carries a high cost of mortality. Group size did not affect the likelihood that non-breeding females (MSM: hazard ratio = 1.003, 95% CI [0.973,1.033]) or breeding females disappeared (MSM: hazard ratio = 1.058, 95% CI [0.970,1.156]). The disappearance of these two classes of females was probably more often the result of in-situ mortality, as we never identified cases where single females or breeding females disappeared from an established burrow system and later settled elsewhere.

### Within-group recruitment

**Figure 4.**
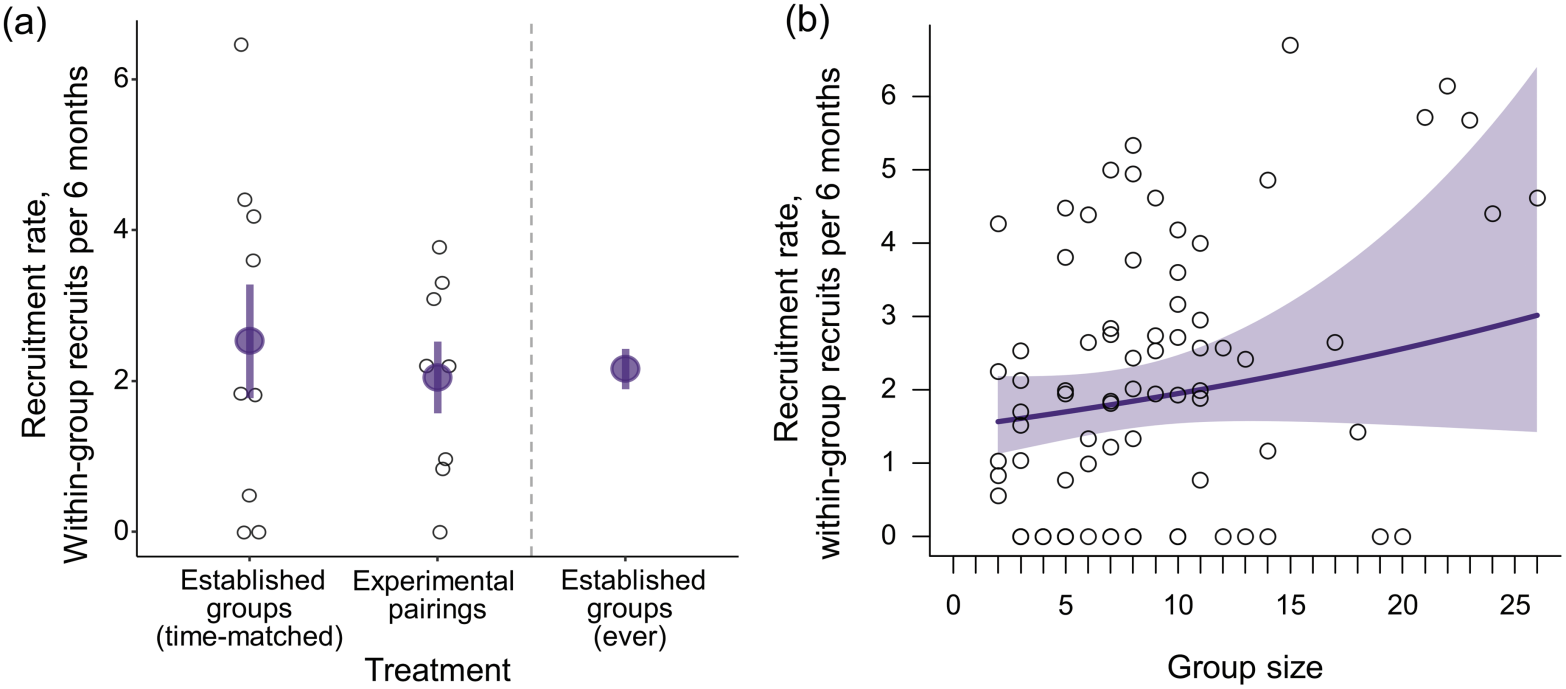
The effect of group size on within-group recruitment. (A) The recruitment rate of established groups (*n* = 9) did not differ from that of newly created pairs (*n* = 8) when measured over the same period. The mean rate of recruitment in this experimental period (middle panel in a) was similar to the overall mean recruitment rate in the population (right panel in a; average of all values in b). The larger purple points denote the treatment mean ± 1 SEM. (B) In contrast, longitudinal analyses of group size (*n* = 78) across the duration of study detected a modest positive effect of increasing group size on the rate of within-group recruitment (0.216 ± 0.106, *z* = 2.04, *p* = .042, GLMM, link-scale); solid line shows the predicted mean and shading shows the 95% confidence intervals. In both panels the black unfilled points give raw data, corrected for the trapping interval duration, and in all cases, recruitment has been standardized to a 6 monthly rate according to the time difference between the first capture and the second capture.

Experimentally created pairs recruited a similar number of offspring into their group as established groups that were captured and recaptured over the same period (Welch’s *t*-test, *t* = 0.253, df = 14.02, *p* = .80; Figure 4a; Supplementary Figure S12 details capture history after pairing). Recruitment rates in experimental and control groups over the experimental period were comparable to the total recruitment rate over the whole study period (Figure 4A), and environmental conditions across the experimental period were typical of those seen in the Kalahari over a longer time span (Supplementary Figure S13).

Across the duration of the study, increasing group size was associated with a modest increase in within-group recruitment rate (GLMM: 0.216 ± 0.106, *z* = 2.04, *p* = .042; Figure 4B and Supplementary Table S6). Changing the group size term to reflect the mean group size across the capture and recapture event did not qualitatively affect the result.

There was an indication that recruitment rates were associated with climatic variation. Increased rainfall in the year prior to the first trapping showed a positive but nonsignificant association with recruitment (geometric mean monthly rainfall, GLMM: 0.159 ± 0.086, *z* = 1.85, *p* = .065). The body mass of the breeding female did not significantly correlate with recruitment (GLMM: −0.090 ± 0.089, *z* = −1.02, *p* = .31). The inclusion of an interaction between rainfall and group size suggested that the effect of group size on recruitment rate did not depend upon recently experienced weather conditions (GLMM: 0.108 ± 0.080, *z* = 1.36, *p* = .17).

### Early-life growth and adult body mass

**Figure 5.**
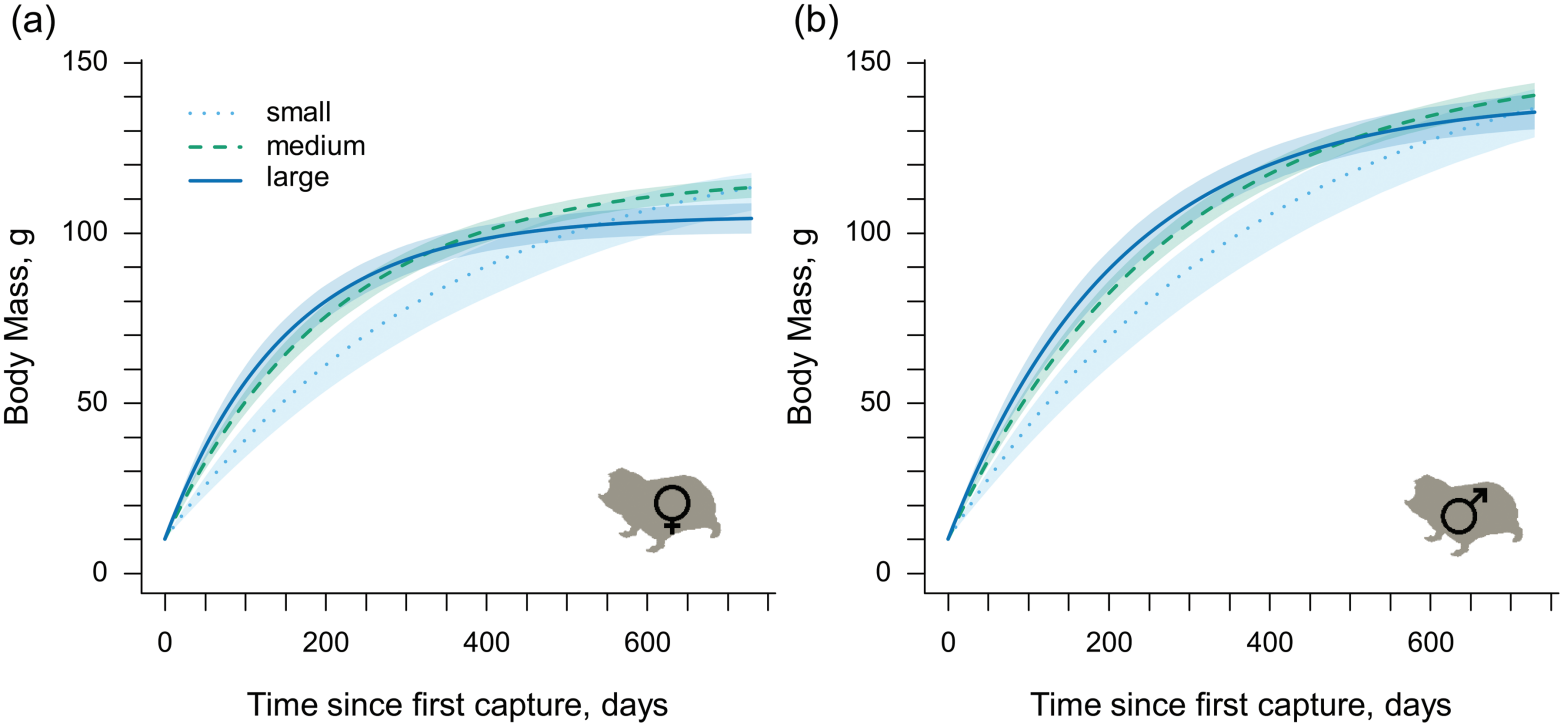
The effect of group size on body mass growth. (A) and (B) display the predicted body mass growth of a male or female mole-rat first captured at 10 g, the average size of a pup at parturition. Body mass growth depended on whether the individual developed in a small (4 individuals, dotted line), medium (12 individuals, dashed line), or large (20 individuals, solid line) group. Curves are derived from von Bertanlanffy interval equations and show the predicted mean body mass, with the shaded areas indicating 95% confidence intervals. Growth curves are estimated from models fitted to 381 mass records taken from female non-breeders in their natal group (193 unique individuals), and 456 mass records taken from male non-breeders in their natal group (214 unique individuals).

Individuals in larger groups showed higher body mass growth in early life, and this effect was present in both sexes (Figure 5 and Supplementary Table S7). However, growth rates slowed earlier and faster in larger groups with the consequence that individuals in larger groups ultimately went on to attain a lower asymptotic body mass (NLMM: male *A*_*GS*_ = −8.10 ± 2.02, *p* < .001; female *A*_*GS*_ = −9.42 ± 1.47, *p* < .001; Supplementary Figure S14, see Supplementary Material for further treatment: Supplementary Figure S15 and Supplementary Table S9). Increasing group size was also associated with lower asymptotic skeletal size, though statistical support for the effect was only present in females (NLMM: male *A*_*GS*_= −0.047 ± 0.036, *p* = .19; female *A*_*GS*_= −0.113 ± 0.027, *p* < .001; Supplementary Table S8). In contrast to the body mass models, there was no support for a relationship between group size and the skeletal growth rate in either sex (Supplementary Table S8).

## Discussion

Naked mole-rats and Damaraland mole-rats have often been cast as extreme examples of mammalian sociality, and it has commonly been stated that successful reproduction depends on help provided by non-breeders. In addition, influential papers have suggested that there is a high degree of similarity between the social systems of mole-rats and those of social insects, and most studies still refer to social mole-rats—or naked mole-rats specifically—as eusocial (Bennett & Faulkes, 2000; Braude et al., 2021; Buffenstein et al., 2022; Burda et al., 2000; Faulkes & Bennett, 2021). However, some of these comparisons have relied extensively on research on captive mole-rats maintained in tube systems that are seldom longer than a few meters and that prevent dispersal—whereas the tunnel systems of natural groups often extend over more than 1 km (Bennett & Faulkes, 2000). This strong focus on captive studies has led to a relatively limited understanding of how new groups form, and of how variation in group size is correlated with relevant measures of fitness in wild populations. Our own records of the dynamics of groups of wild Damaraland mole-rats show that a substantial proportion of breeding females are individuals that have dispersed and settled alone in a burrow system. These solitary individuals display high survivorship and maintain a body condition that is comparable to individuals living in groups, despite foraging alone and lacking access to other putative benefits of group-living, such as the benefits of group defense or social thermoregulation. Once joined by a male, these nascent pairs produce pups at a rate which is similar to that of established groups, even though inexperienced breeders face known developmental costs of reproduction (Johnston et al., 2021). These characteristics of the breeding ecology of Damaraland mole-rats highlight that they should not be considered obligate cooperative breeders—and it is important to appreciate that the similarities between the social system of Damaraland mole-rats and eusocial invertebrates are more limited than previously thought.

While the presence of non-breeding group members was not necessary for successful reproduction by breeders, our study finds that rates of recruitment are higher in larger groups than in smaller ones, and in this respect, it aligns with previous studies of social mole-rats in captivity and in the wild (Houslay et al., 2020; Young et al., 2015). However, the strength of the positive association between group size and reproductive output in our population is modest and the benefits of cooperative foraging may therefore be lower than previously thought. In many other social animals, group size, breeding success, and the growth of individuals increase in groups occupying more productive ranges and habitats so that associations between group size and breeding success do not necessarily reflect causal relationships (Cockburn, 1998; Downing et al., 2020). Breeders in larger groups are also likely to be older and more experienced. Efforts to separate out these competing influences—such as via helper removal experiments—have yet to be carried out on social mole-rats in the wild, and until such work is carried out, the importance of non-breeders for the growth and survival of juveniles and for the fecundity of breeding females may remain uncertain. What is made clear by our study is that unlike in obligatorily eusocial insects and some of the more specialized cooperatively breeding vertebrates where non-breeding individuals contribute substantially to the provisioning of dependent young (Bourke & Franks, 1995; Creel & Creel, 2015; Koenig & Dickinson, 2016; Oster & Wilson, 1978), in Damaraland mole-rats, individuals can survive and breed successfully without assistance from non-breeders.

Instead of being maintained by selection for reproductive cooperation, group-living in mole-rats may be largely explained by ecological constraints on dispersal (Emlen, 1982, 1995). Such constraints have been important in the evolution of group-living in rodents generally (Firman et al., 2020; Solomon, 2003), and while often also depending on phylogeny and other aspects of life history (Smorkatcheva & Lukhtanov, 2014; Sobrero et al., 2014), the low recapture rate of non-breeders in our study supports the view that dispersal carries a high mortality risk for mole-rats. Like others, we find that dispersal is concentrated around intermittent periods of rainfall (Jarvis et al., 1994; Spinks et al., 2000; Torrents-Ticó et al., 2018; Young et al., 2010), when the soil is soft enough to facilitate burrow digging and permit the establishment of new burrow systems. Nevertheless, by following individuals longitudinally and explicitly modeling their life history trajectories, our results provide the strongest evidence to date that most non-breeders disperse from their natal group in the first few years of adulthood—despite the high mortality risk—and probably do so alone in most instances (Finn et al., 2022). The idea that some individuals have adopted an alternative life-history strategy of lifetime philopatry (Burda et al., 2000; Jarvis & Bennett, 1993) is therefore probably incorrect. A strategy of lifetime philopatry is unlikely to be strongly selected for if the indirect fitness benefits of staying group-bound are relatively low—as suggested by our results—and if the opportunities for inheriting a breeding position are limited. In support of the latter argument, territory inheritance was relatively uncommon for both sexes in our study population and, as in other study populations, solitary dispersal was the most frequent route to the acquisition of a breeding position (Torrents-Ticó et al., 2018).

In demonstrating that solitary dispersal can bring reproductive rewards, our study emphasizes that female Damaraland mole-rats acquire breeding positions in ways that differ from many obligate cooperatively breeding vertebrates and from those eusocial invertebrates that found new nests by swarming. In obligate cooperative breeders, the inheritance of breeding positions—including the natal territory and preexisting helpers—is often the commonest route to breeding for the habitually philopatric sex, and if individuals of either sex disperse, then they typically do so in same-sex coalitions (Downing et al., 2018; Kingma, 2017; Maag et al., 2018; Ridley, 2012; Woodroffe et al., 2020). Single dispersers or small coalitions can have difficulty establishing new breeding groups in many of these species and the effects of group size on fitness is often initially strongly positive (Ebensperger et al., 2012; Groenewoud & Clutton-Brock, 2021; Russell, 2004; Solomon & French, 1997). When single queens in obligate and facultative eusocial insects attempt to establish a new colony, group founding is also characterized by extremely high chances of group failure. Failure is commonly reduced when several queens establish new nests together (Bernasconi & Strassmann, 1999; Cahan & Julian, 1999; Chiu et al., 2018; Tibbetts & Reeve, 2003) or when colonies fission into spatially distinct breeding units, as for instance in swarm founding social wasps (Cronin et al., 2013; Nonacs & Reeve, 1995; Queller & Strassman, 1988). In contrast, routes to breeding in mole-rats appear closer to many facultative cooperatively breeding vertebrates where solitary dispersal is the most frequent route to breeding, dispersal coalitions are rare and territory inheritance happens to a varying extent (Dupont et al., 2022; Kingma et al., 2021; Mayer et al., 2017).

The relative rarity of territory inheritance in our study population may be related to the low rate of successful immigration by males into established groups. Most social mole-rat groups are nuclear families and females will not readily mate with their father or brothers (Burland et al., 2004; Cooney & Bennett, 2000). As a result, natal individuals typically lack access to unrelated mating partners and if either breeder dies, all group members may slowly disperse and groups may dissolve (Brainerd et al., 2008; Creel & Creel, 2002; Jarvis & Bennett, 1993; Jarvis et al., 1998; see also Duncan et al., 2021). The fact that females rarely inherit a breeding position might also explain why sex differences in dispersal timing are small in Damaraland mole-rats (this study; Torrents-Ticó et al., 2018) and why males and females invest similarly in cooperative foraging behavior, with neither sex standing to gain more from helping to rear offspring that can later assist in their own reproductive efforts (Clutton Brock et al., 2002; Downing et al., 2018; Russell, 2004).

Overall, our study highlights that the positive effects of non-breeding individuals on breeder reproduction in Damaraland mole-rats have been overemphasized and that the reliance of the breeders on non-breeders in this species may instead be closer to some family-living or facultatively cooperative species where breeders do not rely on the assistance of helpers. Breeders may tolerate non-breeders remaining in their group as a form of extended parental care, even if their retained offspring provide limited fitness benefits (Ekman, 2006; Ekman & Griesser, 2016). While our study is focused on a single population, the environmental conditions and soil characteristics of our site are similar to other locations where longitudinal sampling of Damaraland mole-rats has been conducted and are representative of the general ecological conditions found throughout the species’ range (Supplementary Figures S6-S8 and Supplementary Table S3). Differences in demography and social organization between studied populations also appear to be small, including variation in group size, sex differences in philopatry, and patterns of dispersal (Supplementary Table S2). However, it remains possible that the dispersion of food resources varies spatially and that this modifies the costs and benefits of group-living in different populations. There are also other ways in which group-living might benefit individuals which we have not been able to fully investigate in our population to date. Chief among these is the possibility that larger groups persist for longer than smaller groups (a group allee effect: Jungwirth & Taborsky, 2015; Keynan & Ridley, 2016). Our finding that single females enjoy high survivorship would seem to downplay the importance of group size on group persistence, but the long potential lifespan of Damaraland mole-rats (more than 10 years: Schmidt et al., 2013) might mean that particularly long time series on the order of several decades are needed to critically examine group persistence.

Lastly, our study raises questions about the adaptive importance of group size in naked mole-rat societies. Naked mole-rat groups are typically far larger than Damaraland mole-rat groups, reproductive skew is more pronounced (Bennett & Faulkes, 2000; Faulkes & Bennett, 2016), and contrasts in size and shape between breeders and non-breeders are greater (Dengler-Crish & Catania, 2007). Yet despite being the focus of intense study in captivity there are currently no estimates for the effect of group size on survival and reproduction from natural populations of naked mole-rats, and the relevance of direct versus indirect fitness benefits in shaping individual life history decisions awaits investigation. Though logistically challenging, demographic studies of naked mole-rats can serve as an important reference point against which to compare mole-rat societies to one another (see Braude, 2000) and to other cooperative taxa, and can clarify the extent to which naked mole-rats present an extreme case of vertebrate sociality.

## Supplementary Material

qrad023_suppl_Supplementary_Material

## Data Availability

All data and code are available online (https://github.com/JThor1990/DMR_GroupSizeEffects).
