# Supplementary material for: Damaraland mole-rats do not rely on helpers for reproduction or survival
Source: Evol Lett. 2023 May 29;7(4):203–15. doi: 10.1093/evlett/qrad023 (PMC10355180; doi:10.1093/evlett/qrad023)
Supplement: qrad023_suppl_Supplementary_Material [file qrad023_suppl_supplementary_material.pdf]

Supplementary Material for

Damaraland mole-rat breeders do not rely on helpers for reproduction or survival

Jack Thorley*, Hanna M. Bensch, Kyle Finn, Tim Clutton-Brock, Markus Zöttl

*Corresponding author. Email: jbt27@cam.ac.uk

This PDF file includes:

Supplementary Text
Figures S1 to S15
Tables S1 to S10
References

Contents

Fig S1. The distribution of the Damaraland mole-rat (DMR) and climatic variation at the Kuruman River Reserve (KRR) 3

Longitudinal Sampling at the Kuruman River Reserve (KRR) 4-8
Fig. S2. Trapping locations of Damaraland mole-rat groups 4
Fig. S3. History of trapping effort in and around the KRR 5
Fig. S4. History of trapping effort at KRR, trapping periods 6
Fig. S5. History of trapping effort at "Lonely", with trapping periods 7
Table S1. Summary of captures for each trapping period 8

Demographic, climatic and soil characteristics across DMR study populations 9-14
Table S2. Demographic comparison of three medium to long-term studies 10-11
Fig. S6. Long-term annual variation in rainfall and temperature across study sites 12
Fig. S7. Long-term monthly variation in rainfall and temperature across study sites 13
Table S3. Predicted soil properties of the three medium to long-term study populations 14
Fig. S8. The predicted soil properties of the three study populations compared to soil properties across the DMR distribution 14

Multi-state modelling of life history trajectories 15-18
Fig. S9. Multi-state model for the life history transitions of females, with Q matrix 15
Fig. S10. Life-history trajectories (states) of females 16
Table S4. State-related survivorship of females 17
Fig. S11. The duration of philopatry (timing of dispersal) for males and females 18
Table S5. The effect of rainfall and body weight on the duration of philopatry 18

The body condition of single females 19

Within-group recruitment 20-22
Fig. S12. Group structure and trapping history of groups that were created experimentally through the addition of an unfamiliar male to the burrow systems of single females 20
Fig. S13. The timing of the experimental pairings in relation to climate KRR 21
Table S6. The recruitment rate of offspring into groups, longitudinal analysis 22

Growth and adult body mass 23-28
Table S7. Body mass growth 23
Table S8. Incisor width growth 24
Fig. S14. The rate of change in incisor width and body mass for females 25
Fig S15. Adult body mass of non-breeding males and females 27
Table S9. Adult body mass of non-breeding males and females 28

Analyses Summary 29-30
Table S10. Summary of the analyses carried out in the paper, with data information 29-30

Supporting information references 31

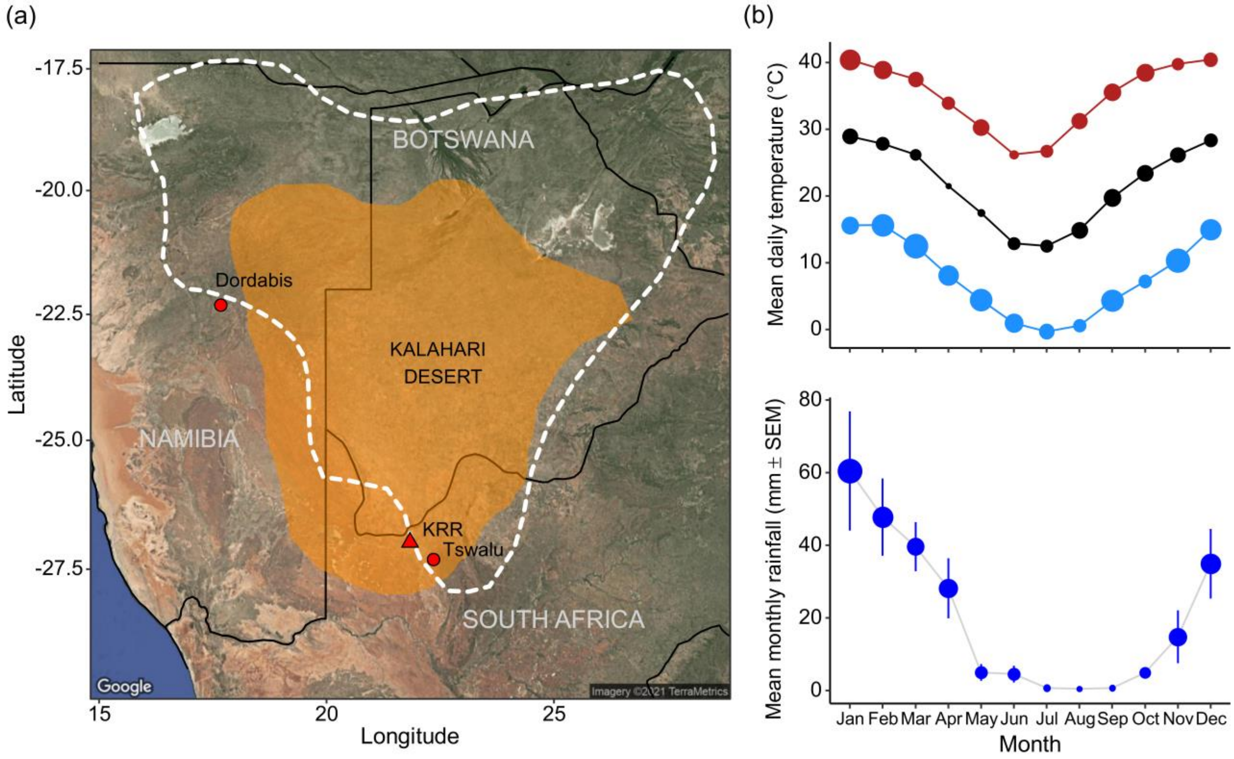

Figure S1. (a) The distribution of the Damaraland mole-rat in sub-Saharan Africa. The predicted current distribution is highlighted by the white dashed line [taken from IUCN, 30th June 2021], with the Kalahari Desert indicated by the shaded polygon. The location of the Kuruman River Reserve (KRR) is also highlighted, as are two other sites where medium to long-term capture-mark-recapture studies of Damaraland mole-rats have been carried out. **(b) The climate at the KRR between 2010 and 2020**. Upper panel shows the mean, minimum, maximum daily temperature in each month, averaged across the years, and the lower panel shows the total monthly rainfall (± SEM), averaged across years; points are scaled to 1SEM. Climate data taken from NASA's GMAO MERRA-2 assimilation model and GEOS 5.12.4 FP-IT.

Longitudinal sampling at the Kuruman River Reserve

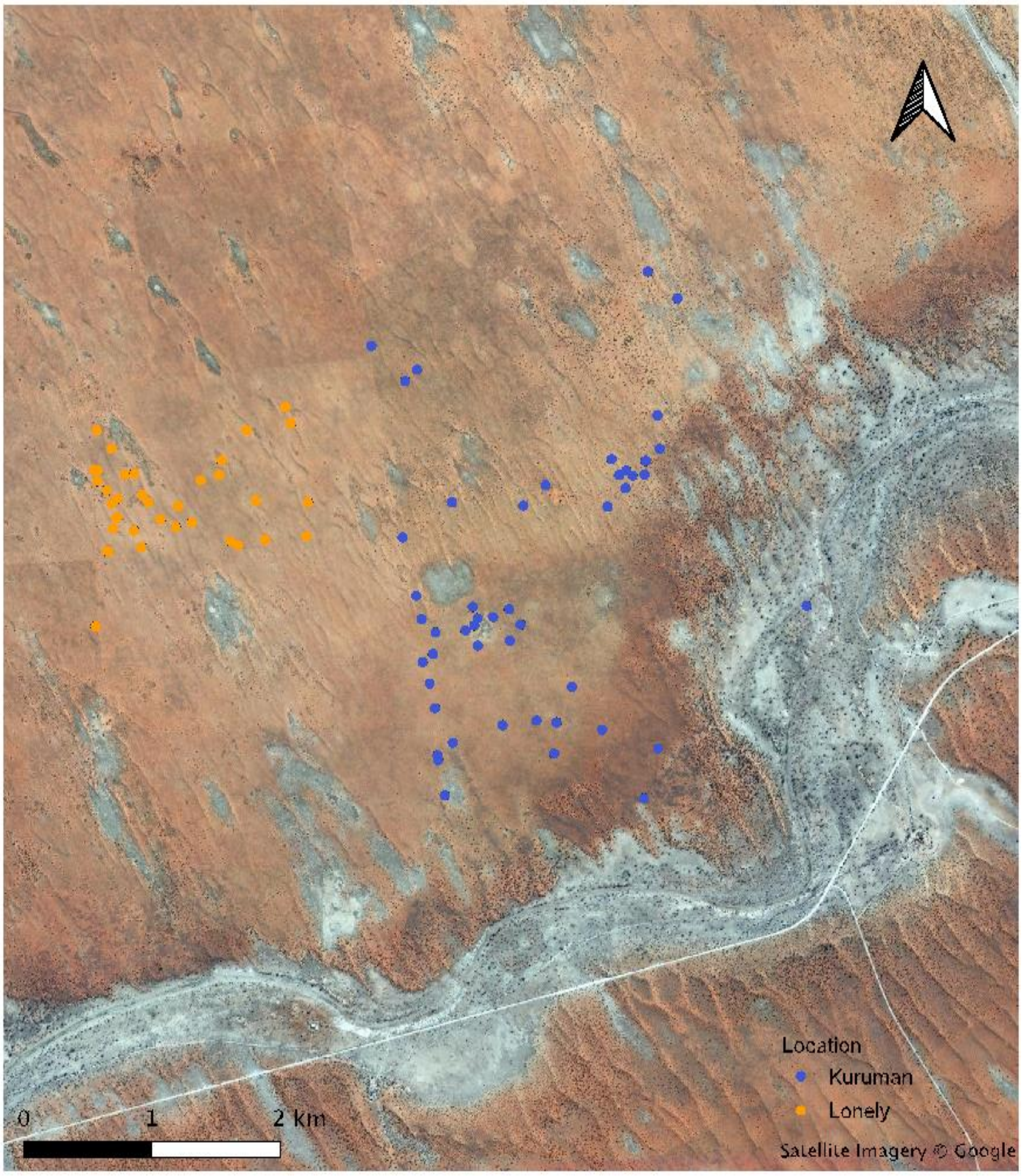

Figure S2. The trapping locations of Damaraland mole-rat groups captured at the Kuruman River Reserve and Lonely farm between 2013 and 2020. Each group is noted by a point which marks the centroid of all group captures, with the point colour corresponding to location, "Kuruman" or "Lonely". Note that with only one exception, mole-rat groups were distributed entirely within the red 'arenosol' soils, where their principal food (Gemsbok cucumber) is found. The one exception to this pattern was a single dispersing female who temporarily burrowed in an area of calcareous soils, which appear light grey from satellite. These calcareous soils highlight the path of the long dried up Kuruman river as it passes through the reserve.

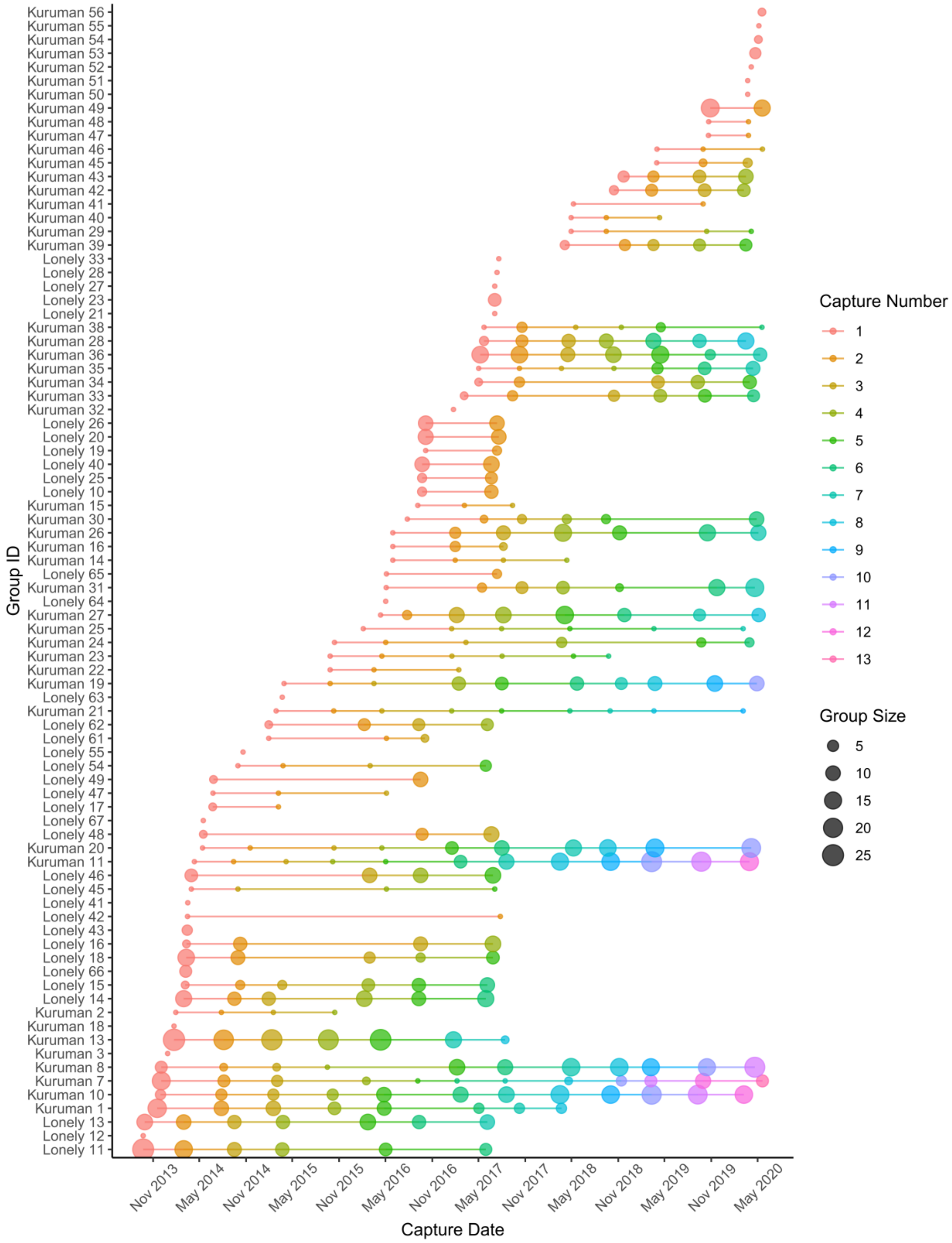

Figure S3. The history of trapping effort of Damaraland mole-rat groups in and around the Kuruman River Reserve. Each unique group has its own row, where successive capture events are denoted by points moving right along the x-axis. The size of the points is proportional to the size of the group at each capture event.

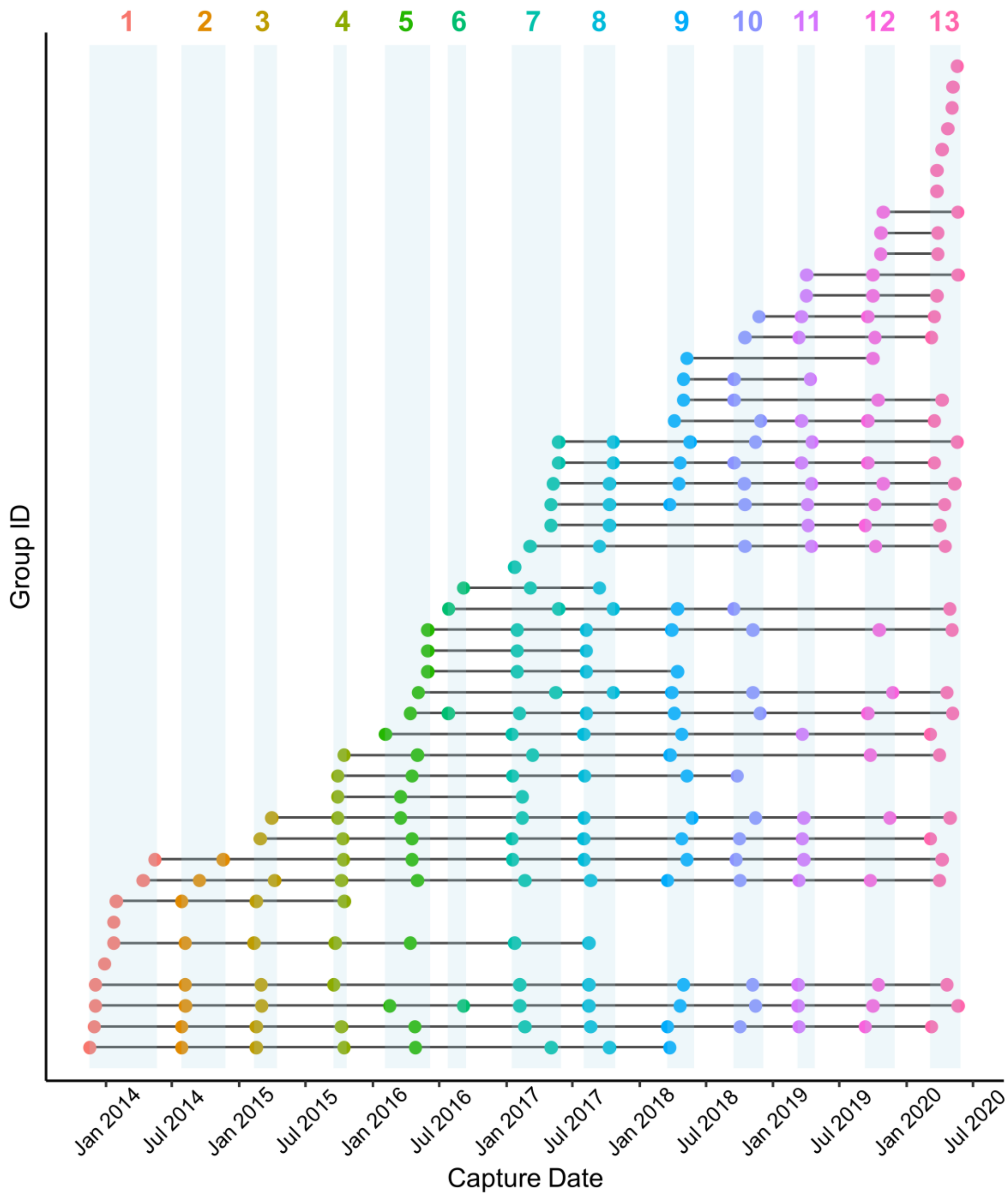

Figure S4. The history of trapping effort for Damaraland mole-rat groups at the Kuruman River Reserve in the Kalahari, separated into discrete trapping windows. Periods are defined by any break in trapping effort of more than 2 months. By doing so, no group is captured twice within a trapping period. Also, most groups are captured in successive trapping periods, with very few groups ‘skipping’ trapping windows- except for period 6, where only a few groups were recaptured. In the few other cases where groups skip a trapping period, this is because no mounds were visible throughout the period, and when this occurs, it is not known whether a group had collapsed/disappeared or was present underground but not digging.

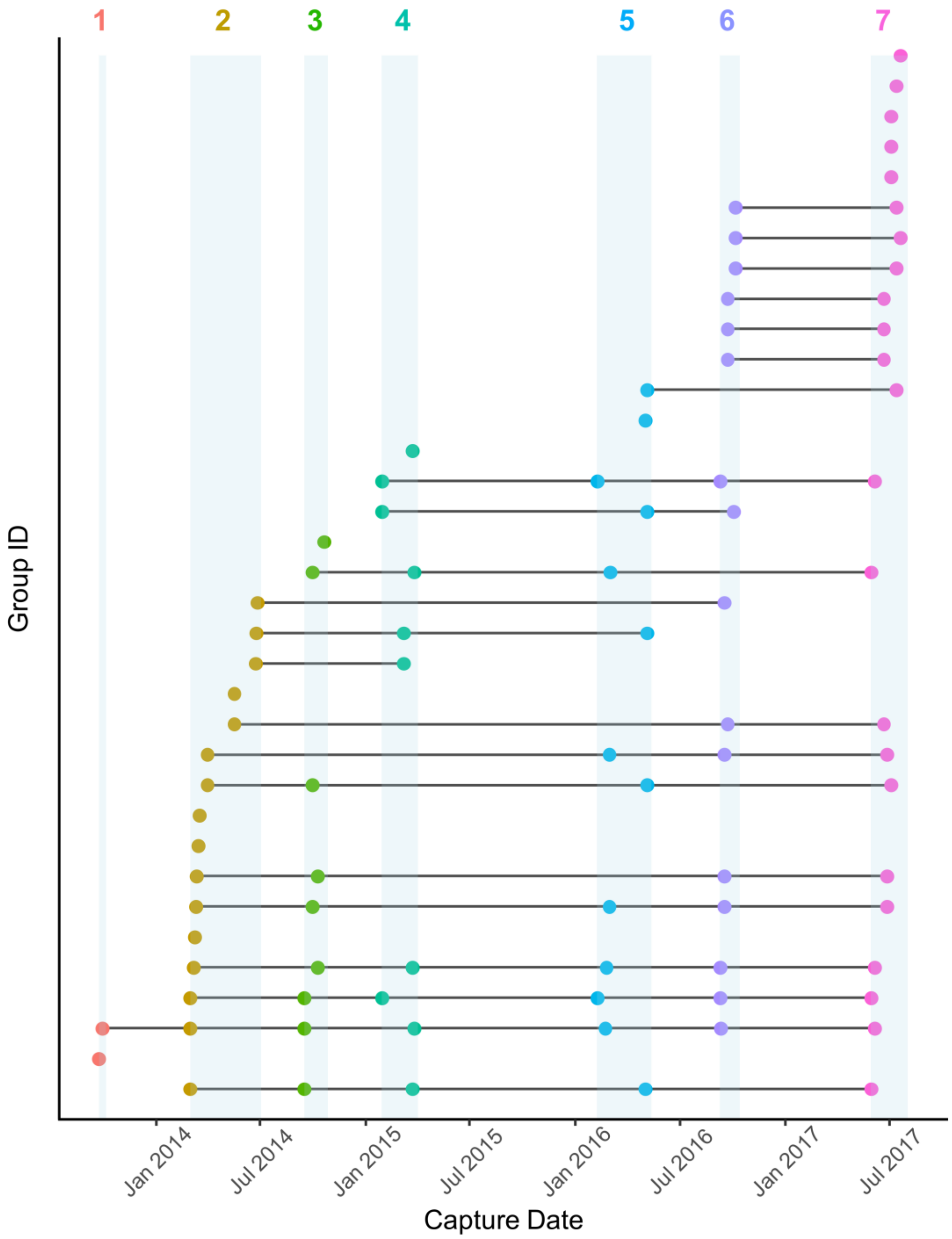

Figure S5. The history of trapping effort for Damaraland mole-rat groups at "Lonely" in the Kalahari, separated into discrete trapping windows. Periods are defined by any break in trapping effort of more than 2 months. By doing so, no group is captured twice within a trapping period.

Table S1. Summary of captures for each trapping period. Recapture rate was calculated as the number of recaptured individuals divided by the total number of individuals captured in the same trapping period. By the end of the first trapping period, two individuals had already been recaptured within new groups (hence 2.6 % recapture rate). Mean recapture rate (N recaptures / N captures within a trapping period, excluding the first trapping period) at Kuruman was 73.1 %, and 54.8% at Lonely.

**Location**	**Trapping Period**	**Period Start**	**Period End**	**N Groups**	**N Males**	**N Females**	**N Ind**	**N New Ind**	**N Recap Ind**	**Recap %**
Kuruman	1	2013-11-17	2014-05-21	10	35	41	76	74	2	2.6%
Kuruman	2	2014-07-27	2014-11-24	8	19	28	47	9	38	80.9%
Kuruman	3	2015-02-10	2015-04-14	9	21	28	49	7	42	85.7%
Kuruman	4	2015-09-16	2015-10-22	12	13	30	43	8	35	81.4%
Kuruman	5	2016-02-04	2016-06-06	17	20	38	58	15	43	74.1%
Kuruman	6	2016-07-25	2016-09-12	4	2	4	6	1	5	83.3%
Kuruman	7	2017-01-16	2017-05-30	27	68	51	119	68	51	42.9%
Kuruman	8	2017-07-31	2017-10-26	24	67	61	128	40	88	68.8%
Kuruman	9	2018-03-17	2018-05-30	24	79	70	149	46	103	69.1%
Kuruman	10	2018-09-14	2018-12-04	22	71	68	139	34	105	75.5%
Kuruman	11	2019-03-09	2019-04-24	20	83	75	158	41	117	74.1%
Kuruman	12	2019-09-09	2019-11-30	24	93	97	190	65	125	65.8%
Kuruman	13	2020-03-05	2020-05-27	35	114	128	242	60	182	75.2%
Lonely	1	2013-09-23	2013-10-06	3	20	18	38	38	0	0.0%
Lonely	2	2014-03-01	2014-07-03	17	40	45	85	58	27	31.8%
Lonely	3	2014-09-16	2014-10-27	9	19	29	48	13	35	72.9%
Lonely	4	2015-01-29	2015-04-02	10	14	20	34	9	25	73.5%
Lonely	5	2016-02-08	2016-05-12	13	24	41	65	30	35	53.8%
Lonely	6	2016-09-09	2016-10-13	16	55	54	109	66	43	39.4%
Lonely	7	2017-05-30	2017-08-02	24	85	72	157	67	90	57.3%

Demographic, climatic and soil characteristics across DMR study populations

Including our own study population there have been two other medium to long-term studies of wild Damaraland mole-rats that provide some comparable demographic information across a large number of sampling periods (multiple group captures across multiple years). This includes at Dordabis, Namibia (e.g., Jarvis et al., 1998; Young et al., 2015; Torrents Ticó et al., 2018), and at Tswalu, South Africa (e.g., Young & Bennett, 2010; Finn et al. 2018; Mynhardt et al., 2021). There have also been shorter studies conducted elsewhere, including in Hotazel, South Africa (e.g. Burland et al., 2002), Waterberg, Namibia (e.g. Burland et al., 2002), and in the Kgalagadi Transfrontier Park (e.g. Lovegrove & Knight-Eloff, 1988; Jarvis et al., 1998).

Here, we briefly collate some basic features of the social organisation and demography of the three study populations in order to place our own population/study into wider context. We also compare the climate and soil characteristics of the populations, as one of the most pervasive arguments for the evolution of sociality in the African mole-rats is the aridity-food distribution hypothesis, which proposes that mole-rat sociality evolved in environments with reduced rainfall, which impacts soil hardness and raises the energetic costs of digging while also being associated with clumped food resources. In this environment, natal philopatry and cooperative foraging has been argued to increase the efficiency of locating food resources (e.g., Faulkes et al., 1997; Jarvis et al. 1998; Bennett & Faulkes, 2016). Comparisons of climate, soil, and demography across populations and across species can therefore be used to examine the generality of the aridity-food distribution hypothesis, while also helping to understand the drivers of sociality in mole-rats more generally (e.g., Faulkes et al., 1997; Spinks et al. 2000; Lövy et al. 2012); and may shed light on any contrasts among populations.

Basic demographic comparisons

(See Supplementary Table S2 on next page)

Table S2. Comparison of some basic demographic features of three Damaraland mole-rat populations that have been the focus of medium- to long-term study. Note that trapping at the Kuruman River Reserve is ongoing.

Study Population	Coordinates	Capture effort: group captures	Group size* (Mean ± 1SD)	Sex-bias in philopatry	Male dispersal***	Female dispersal	Group fissioning**	Principal food
Kuruman River Reserve<br>(South Africa)	21.83246, -26.97856	>300<br>(2013-2020)<br>7.5 yrs	8.54 ± 5.32[a]<br>(2-26)	None[a]<br>(estimated ~ 75 days longer than males but not sig.)	Commonly alone; limited evidence of dispersive male coalitions. Immigration into tunnels systems of single females common. Immigration into established groups infrequent. Capture of single males very rare.	Commonly alone; limited evidence of dispersive female coalitions. Most cases involve the establishment of new burrow systems singly; single females common.	Group fission events as defined are rare.	Gemsbok cucumber
Tswalu<br>(South Africa)	22.26670, -27.43337	>200<br>(2004-2006, 2013-2016)<br>6 yrs	6.27 ± 3.86<br>(2-24)[b]<br><br>8.96 ± 5.30<br>(2-24)[c]	Not explicitly tested	2004-2006: "*Males were found to disperse more frequently into established colonies (32% of male dispersal events [7/19])*"[b]<br><br>2013-2016: "*Male-biased immigration rate to established colonies at Tswalu (n = 11:4), [but] the observed rate was not significant*"[c].	2004-2006: "*Female dispersals were typically associated with establishment of new colonies (e.g. colony fission events), with only 12% of dispersal events [2/17] involving immigration into neighbouring colonies*"[b].<br><br>2013-2016: 13.6% (3/22) of immigrants into established groups were females[c].<br><br>Presence of single females remarked upon[b,c,d] (see Figure 5[b]). Less common than at KRR, but not uncommon.	Group fission events as defined are rare.<br><br>Mynhardt et al. 2021[b] identified one case of group fission between 2004-2006, Finn's (2017) data from 2013-2016 noted "*one possible case [of territory budding in Tswalu].*"	Gemsbok cucumber,<br><br>Eland bean[c]
Dordabis<br><br>(Namibia)	17.68364, -22.96667	>500<br>(1988-2002)<br>14 yrs	11.29 ± 6.26[e]<br>(2-41)	Female[f],<br><br>(estimated ~ 40 days longer than males)	74% of males (69/93) dispersed to establish new groups, 26% (24/93) immigrated into established groups. Of all events, 23% dispersed as a coalition** (21/93), all others dispersed alone[f].<br><br>Unclear from tables whether the establishment of new groups is related to males joining single females, or vice versa.	87% of females (52/60) dispersed to establish new groups. Of all events, 12% dispersed as a coalition** (7/60), all others dispersed alone[f].<br><br>Unclear from tables whether the establishment of new groups is related to males joining single females, or vice versa. The presence of single individuals in tunnel systems has been remarked upon earlier in the study[g] but was suggested as uncommon.	Group fission events are rare: "although dispersal coalitions were rare, males were more likely to disperse with other individuals of the same sex than females"[f].<br><br>Previously, it has been stated that "*on the death or experimental removal of the reproductive, the colony fragments, the non-reproductive adults become sexually active and new colonies are founded.*"[g]	Wild onions (Dipcadi spp.)[h]

* All group size estimates calculated for 'groups' with >= 2 individuals.

** Also referred to as 'territory budding'. Defined as multiple individuals "budding" from their current group, either by establishing an adjacent tunnel system or blocking off an existing portion of their tunnel system. As fission events necessarily occur over short distances, it may be difficult to distinguish fission events from coalition events, so here we effectively use the terms as synonyms. It is also unclear in all populations how many coalition events represent true coalitions where individuals dispersed together as a unit, rather than separate episodes of emigration and immigration from the same original tunnel system.

*** Genetic studies of various other *Fukomys* populations supports the general view that male immigration into established groups sometimes takes place (sometimes contributing to multiple paternity within a group), whereas female immigration is mostly absent (e.g. Burland et al. 2004, Patzenhauerová et al. 2013).

a- This study. See also Finn K.T (2021) Potential use of a magnetic compass during long-distance dispersal in a subterranean rodent. *J Mammalogy*, 102:250-257.

b- Mynhardt, S., Harris-Barnes, L., Bloomer, P., Bennett, N.C. (2021) Spatial population genetic structure and colony dynamics in Damaraland mole-rats (*Fukomys damarensis*) from the southern Kalahari. *BMC Ecology and Evolution*, 21: 221.

- Mean group size for the 2004-2006 period at Tswalu is provided in Fig.5 of Mynhardt et al. 2021: 6.33 ± 3.87 (Mean ± 1SD, range = 2-24).

c- Finn, K.T. (2017) Density-dependent effects of body size, philopatry, and dispersal in the Damaraland mole-rat (Fukomys damarensis). MSc Thesis, University of Pretoria. Finn (2017) covers the period from 2013-2016. Mean group size for the 2004-2006 period at Tswalu is provided in Fig.5 of Mynhardt et al. 2021.

d- Young et al. (2010) Physiological suppression eases in Damaraland mole-rat societies when ecological constraints on dispersal are relaxed. *Hormones and behaviour*, 57:177-183.

e- Bennett, N.C., & Faulkes, C.G. (2000) African mole-rats: ecology and eusociality. Cambridge, UK: Cambridge University Press. Calculated from the frequency distribution of group captures in Fig 4.2, page 91, based on 111 complete group captures.

f- Torrents Ticó, M., Bennett, N. C., Jarvis, J. U. M., & Zöttl, M. (2018). Sex-differences in timing and context of dispersal in Damaraland mole-rats (Fukomys damarensis). *J. Zoology*, 306: 252-257.

g- Jarvis, J.U.M., & Bennett, N.C. (1993) Eusociality has evolved independently in two genera of Bathyergid mole-rats – but occurs in no other subterranean mammal. *Behavioural Ecology and Sociobiology*, 33: 253-260.

h- Jarvis, J.U.M., Bennett, N.C., & Spinks, A.C. (1998) Food availability and foraging by wild colonies of Damaraland mole-rats (*Cryptomys damarensis*): implications for sociality. *Oecologia* 113: 290-298.

Climate comparisons

To compare the rainfall and temperature of the three study sites we extracted daily climate data from taken from NASA's GMAO MERRA-2 assimilation model and GEOS 5.12.4 FP-IT (https://power.larc.nasa.gov/data-access-viewer/) from January 1982-December 2020. For temperature, the daily maximum, minimum and mean temperature (ºC) were extracted, while for rainfall we took the total daily precipitation (mm).

On average, our field site in the Kalahari has experienced lower total annual rainfall than both Tswalu and Dordabis over this period (Supplementary Figure S6a, ANOVA, $F_{2,114} = 8.11$, $p < 0.001$. Tukey contrasts both $p < 0.003$ (t-values > 3.39)), while also being hotter, with a higher mean daily maximum temperature across years (Supplementary Figure S6b, ANOVA, $F_{2,114} = 31.40$, $p < 0.001$; Tukey contrasts t- $p < 0.001$ (t-values > 5.07)). Post-hoc testing of model contrasts showed that Dordabis has also been hotter than Tswalu overall (t-value = 2.74, p = 0.019), such that temperature follows a gradient whereby Tswalu < Dordabis < KRR. Supplementary Figure S7 displays the monthly averages for each site across the same 1982-2020 period.

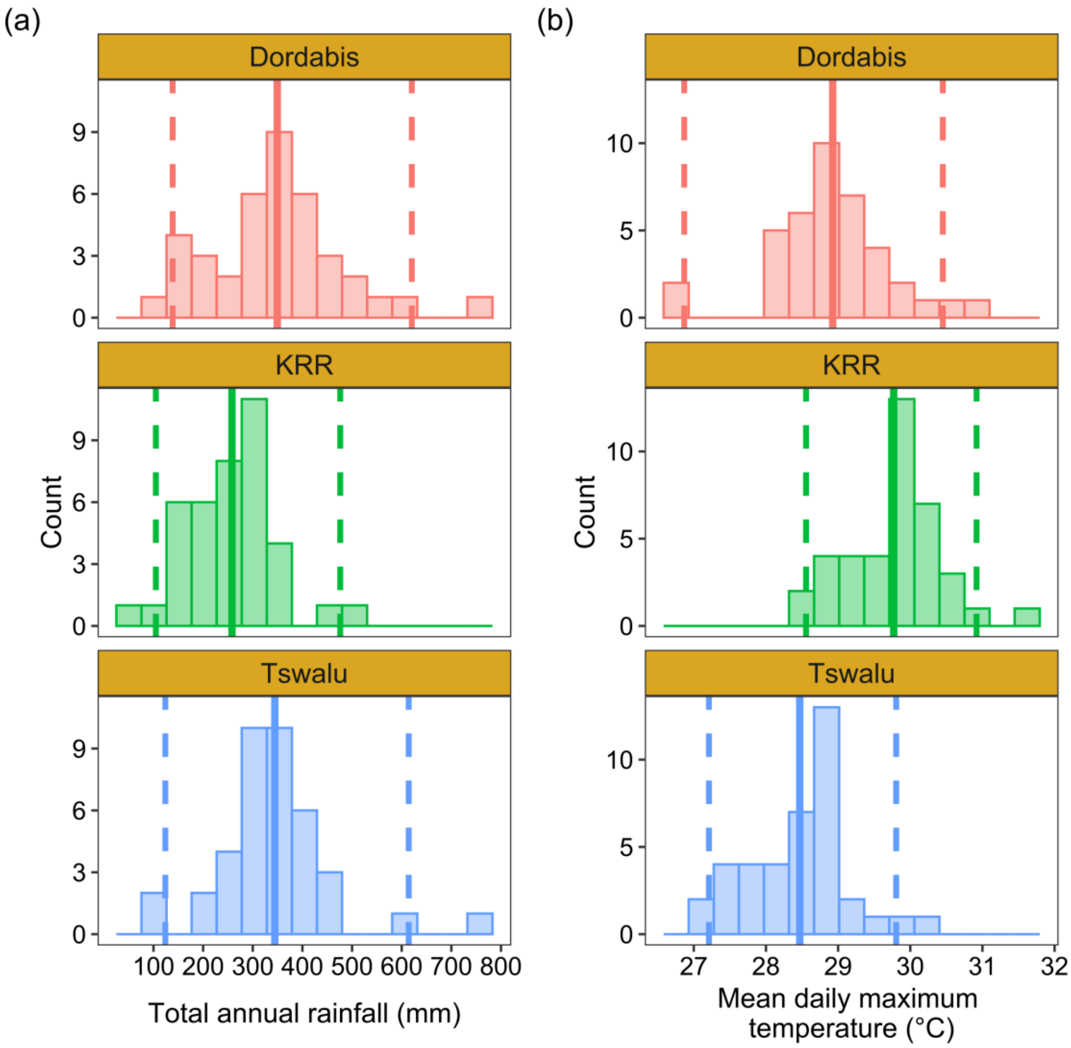

Figure S6. Long-term variation in annual rainfall and temperature across the three medium to long-term study sites. Histograms present the total annual rainfall (a) and mean daily maximum temperature (b) between 1982-2020. The overall mean and 95% confidence intervals are given by the solid and dotted lines, respectively.

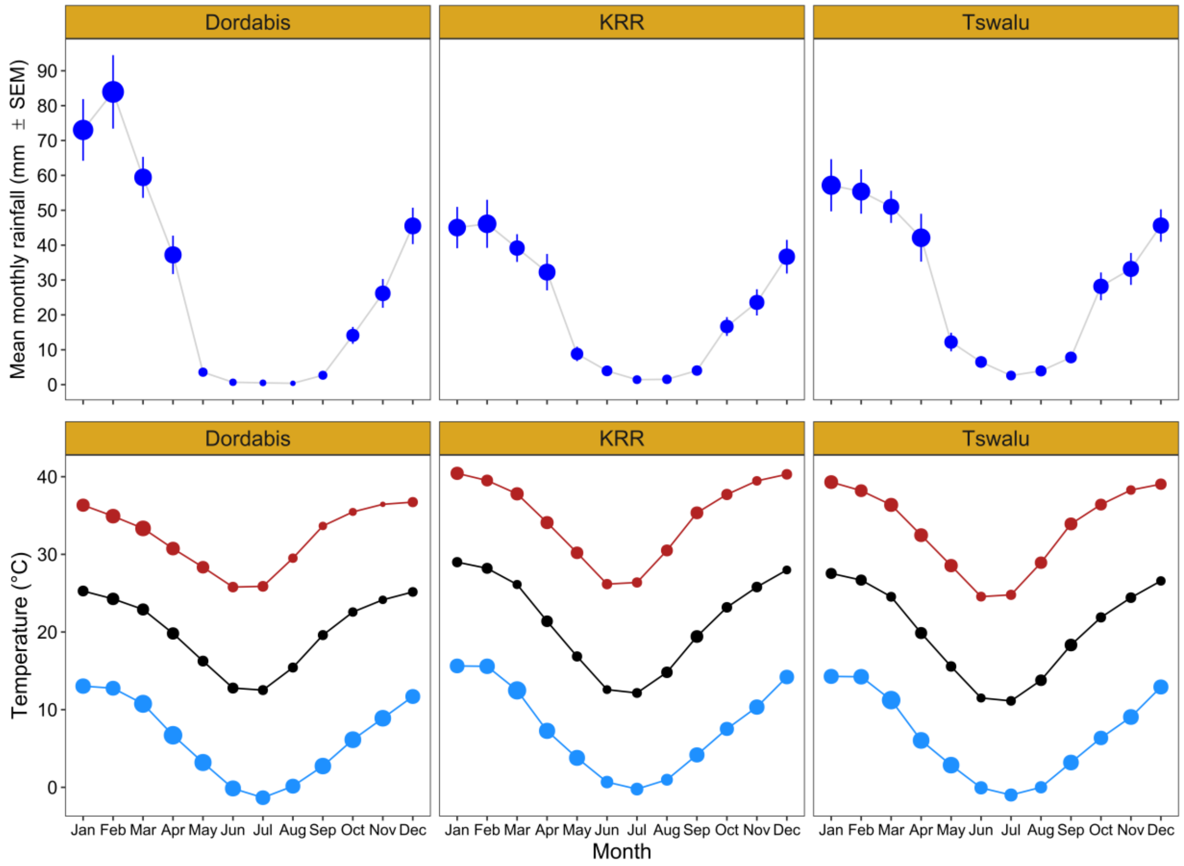

Figure S7. Long-term variation in monthly temperature and rainfall across the three medium to long-term study sites between 1982-2020. Upper panel shows the total monthly rainfall (± SEM), and the lower panel shows the mean, minimum, maximum daily temperature in each month, both averaged across the years.

Soil comparisons

Soil properties for the three field sites were taken from the *SoilGrids* database (Hengel et al. 2017), a global predictive map of soil composition, via the *soilDB* package in R (Beaudette et al. 2022). For each study site, the predicted soil composition was extracted for the focal coordinate, as well as for the vertices of a 2km-by-2km bounding box centred on the focal coordinate. Five data points were used to lower the risk of a single coordinate providing a misrepresentative [predicted] sample. *SoilGrids* provides data at 250m-by-250m resolution for a range of soil depths, and we here averaged all values within the first 100cm of soil as this covers the typical depth of Damaraland mole-rat burrow systems (qualitatively unchanged if using shallower depth profile (< 60cm).

The three study sites have very similar levels of sand in the soil, supporting field observations (Supplementary Table S3). Other soil properties were also very similar, suggesting that all else being equal, the workability of the soil across the three sites is likely to be very similar.

Table S3. Predicted soil properties of the medium to long-term DMR study populations.

Study Population	Bulk density of fine earth fraction (kg/dm^3)	Volumetric fraction of coarse fragments (vol %)	Sand (%)	Silt (%)	Clay (%)	Soil organic carbon (g/kg)	Nitrogen (g/kg)
KRR (South Africa)	1.45	15.0	69.0	14.1	17.0	4.15	0.54
Tswalu (South Africa)	1.45	15.0	69.8	13.6	14.1	3.58	0.52
Dordabis (Namibia)	1.45	13.8	70.2	13.9	15.8	3.47	0.52

We also visually compared the soil composition of the three study sites to the predicted soil composition across the Damaraland mole-rat range. To do so, we generated a grid of locations spaced at regular 50km intervals throughout the principal range of the species (downloaded from IUCN red list, [5th December 2022], Supplementary Figure S8a; https://www.iucnredlist.org/species/5753/22185003#geographic-range) and performed a principal component analysis (PCA) on the seven soil properties, Supplementary Figure S8b. The plotting of the first two principal components shows that the three study sites have [predicted] soil profiles that are representative of the wider Damaraland mole-rat distribution.

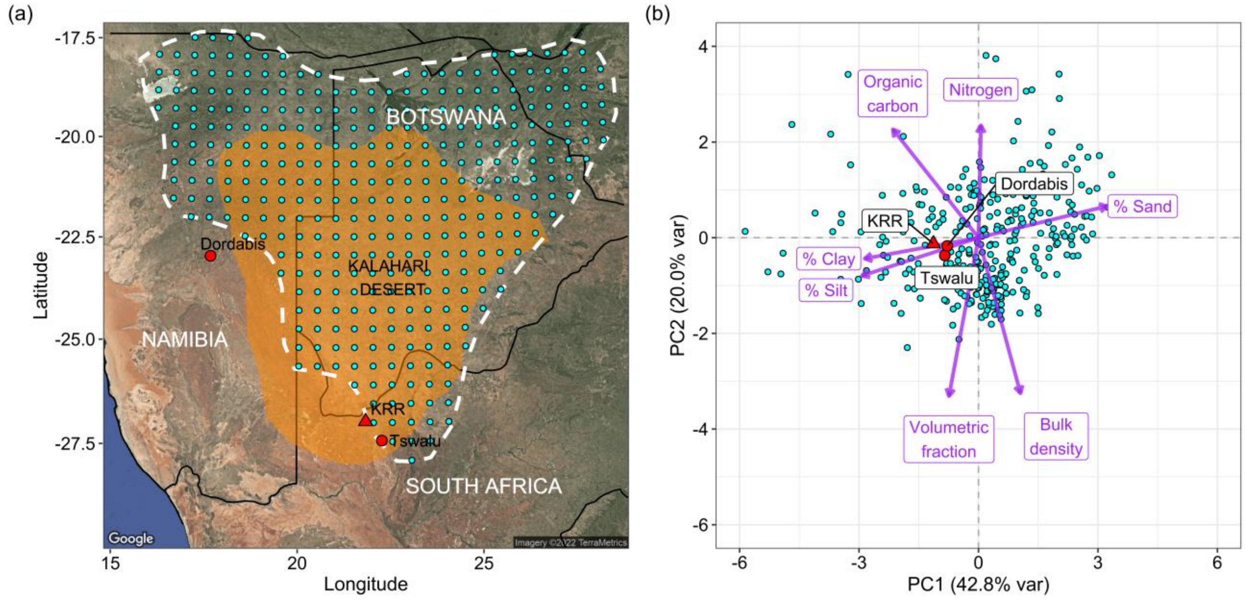

Figure S8. The predicted soil properties of the three medium- to long-term Damaraland mole-rat study populations, compared to soil properties across the Damaraland mole-rat distribution. (a) Highlights the three study locations, in addition to 349 locations samples across the distribution, which is bordered by the white dotted line. (b) Results of the PCA which was used to visual the variation in soil properties across all locations. Points correspond to each location with respect to the first two components, and the factor loadings of the seven soil properties are given by the purple arrows.

Multi-state modelling of life history trajectories

The life history trajectories of individuals were estimated using multi-state Markov models (MSM). Specifically, Markov models were used to:

a) Estimate sex differences in the timing of natal dispersal (Supplementary Table S5)
b) Estimate the survivorship of females in different life history states/stages (Supplementary Table S4)

We first outline the multi-state model used to estimate female survivorship (b), as the philopatry models (a) are a simplified [two-state] version of the former (applied to a data set with both sexes). The rationale for using the multi-state model is described in detail in the Materials and Methods and relies on the assignment of females as either (i) an in-group non-breeder, (ii) a dispersed single individual, (iii) a breeder, or (iv) died/dispersed- all using information on capture histories (Supplementary Figure S10).

The multi-state model can be shown diagrammatically as:

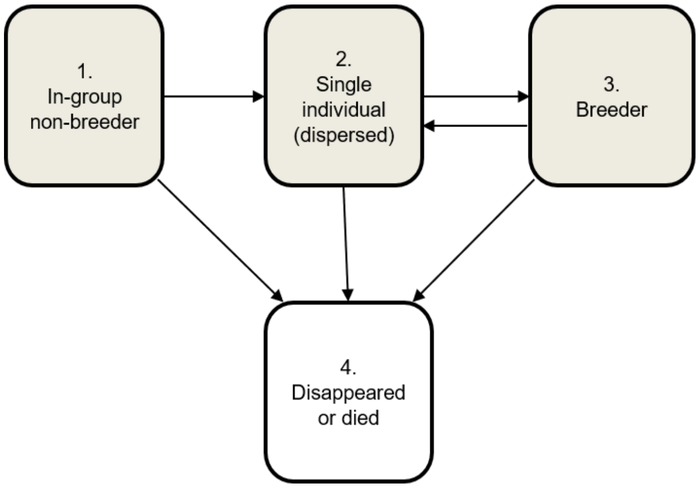

$$Q = \begin{pmatrix} q_{11} & q_{12} & 0 & q_{14} \\ 0 & q_{22} & q_{23} & q_{24} \\ 0 & 0 & q_{33} & q_{34} \\ 0 & 0 & 0 & 0 \end{pmatrix}$$

Figure S9. Multi-state model for the life history transitions of female Damaraland mole-rats, with associated Q matrix. In a small number of cases where individuals were experimentally paired (i.e., given a male when formerly present as a single female and analysed in other papers, e.g., Thorley et al. 2018 Proc. R. Soc. B, 285: 20180897), they were also right censored at the point of pairing so that any transitions from single female to breeder did not include this experimental component. In all cases, the multi-state model was parameterised so that the timing between transitions was not known exactly but was estimated under Markov assumptions- this includes the timing of death/disappearance.

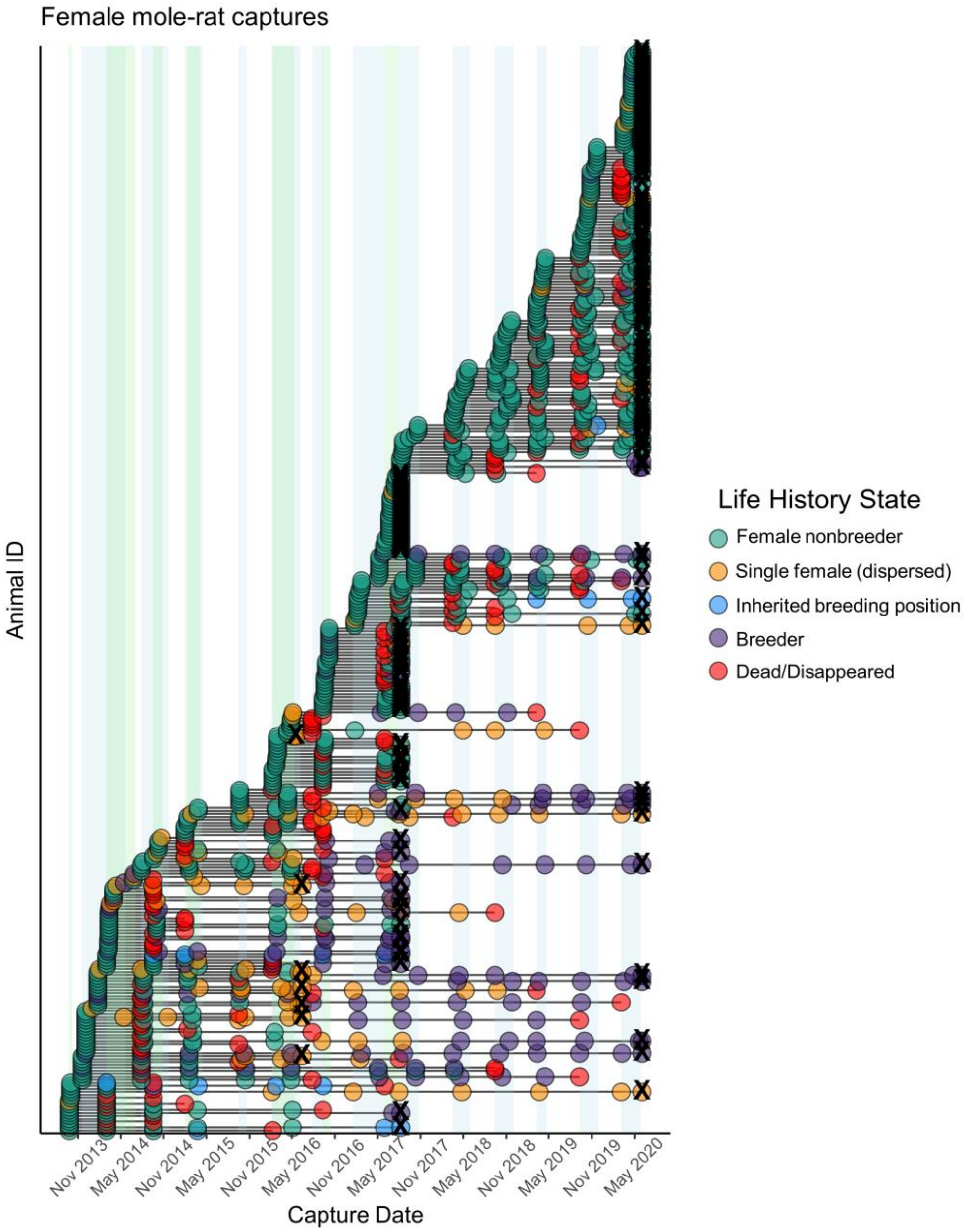

Figure S10. Life-history trajectories (states) of female Damaraland mole-rats captured from 2013-2020. Though analysed together, the fate/current state of female non-breeders at the Kuruman River Reserve and Lonely were assessed separately since they took advantage of different trapping windows (KRR- blue shaded areas; Lonely- green shaded areas). Individual states at any given time are female non-breeder, single female (who has dispersed from her natal group), out-of-natal group breeder (again dispersed) and disappeared/died. In a small number of cases females also inherited the breeding position in their natal group (when an immigrant male had moved in previously). For individuals that were not recaptured at some point before the final trapping period at either location, it was assumed that the individual had died or disappeared at some point between their last capture and start of the next trapping window - as handled by the models. Additionally, individuals captured in the last trapping period at each location were assumed to have persisted in their current state up until the end of the period, where they were then right-censored (**X**).

Table S4. State-related survivorship of females. Probability of transitioning between states over a one-year and a two-year period. Transition probabilities estimated from a multi-state Markov model fitted to all females that were captured across the duration of the study. n = 362 females. Estimates related to survival probability are filled in grey. Confidence intervals estimated using the delta method.

Transition	1 year probability (95% CI)	2 year probability (95% CI)
in-group non-breeder →in-group non-breeder	0.525 (0.466, 0.568)	0.276 (0.221, 0.323)
in-group non-breeder → single female	0.069 (0.047, 0.097)	0.078 (0.051, 0.110)
in-group non-breeder → breeder	0.048 (0.031, 0.077)	0.079 (0.055, 0.115)
in-group non-breeder → dead/disappeared	0.358 (0.314, 0.411)	0.567 (0.513, 0.624)
single female → single female	0.612 (0.469, 0.716)	0.375 (0.217, 0.506)
single female → breeder	0.181 (0.104, 0.312)	0.267 (0.155, 0.404)
single female→ dead/disappeared	0.207 (0.131, 0.318)	0.358 (0.249, 0.512)
breeder → single female	-	-
breeder → breeder	0.867 (0.777, 0.921)	0.752 (0.600, 0.849)
breeder → dead/disappeared	0.133 (0.079, 0.223)	0.248 (0.151, 0.400)

Dispersal timing/Duration of philopatry

We adopted a similar state-based framework to investigate sex differences in the timing of natal dispersal. In order that the timing of dispersal could be approximately related to age, these analyses focussed on individuals that were first captured in their group at less than one year of age (<80g for females, <100g for male; Figure 5 main text). Unlike the female survivorship model, where individuals were assigned to one of two states, the philopatry model defined only two states for males and females: i- *non-breeder* in their natal group, and ii- *disappeared*/*dead/known disperser*. Any conditions under which an individual first left their natal group or disappeared was specified as state (ii). Males and females were modelled together, and a covariate for sex was included so that the transition intensity of males was estimated relative to females. Covariates of weight and rainfall were also included to infer whether the disappearance of non-breeders from groups was mostly due to dispersal or to death in situ; if dispersal was the principal reason for disappearance, then we expected that heavier (and older) individuals would be more likely to disappear [or be successfully recaptured out of their natal group]. We also predicted that increases in rainfall were associated with higher rates of disappearance, which we reasoned would also be suggestive of dispersal rather than death in situ. Rainfall was characterised as the total rainfall (mm) which fell three months prior to leaving state (i).

This model indicated that males and females disappeared from their natal group at a similar age (Supplementary Figure S11). For individuals first captured at less than one year of age, 39.5% of females were predicted to have disappeared one year later (95% CI = [33.5,45.5]), as compared to 43.5% of males (95% CI = [38.3,49.3]). This sex bias reflected a tendency for males to leave their natal group slightly earlier than females, though this effect was not statistically significant (MSM: hazard ratio of males relative to females = 1.125, 95% CI = [0.864,1.476]).

The mean timing of dispersal for males and females that is presented in the main text was estimated as the mean sojourn time in the natal group (*sojourn.msm()* function), when rainfall and body mass were held at their mean value across the data set. For males, the mean sojourn time in the natal group was 1.80 years (95% CI [1.50,2.15]) after their first capture, and for females was 2.02 years (95% CI [1.66, 2.47]). With groups being trapped at roughly 6-month intervals, these values reflect an underestimate of the average age at dispersal.

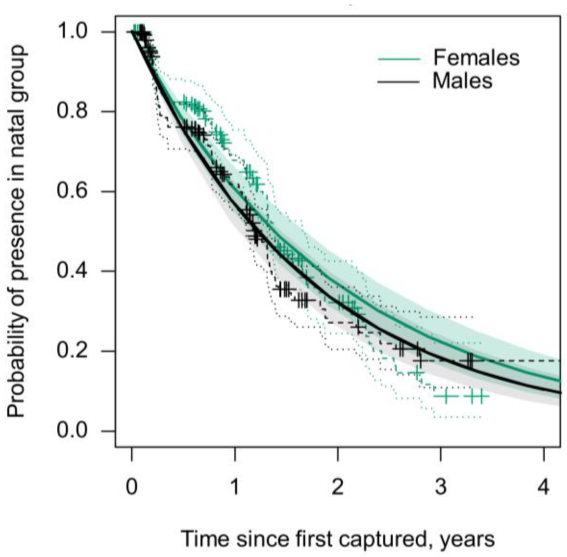

Figure S11. The duration of philopatry (timing of dispersal) for males (black) (green) and females. Solid lines display the expected probability of recapturing non-breeding individuals within their natal group, as estimated from a Markov model fitted to all individuals first captured at less than one year of age (females <80g, males < 100g). Dotted lines display the 'empirical' Kaplain-Meier estimate of survival probability, with crosses denoting cases of censorship. Disappearance combines cases of in-group mortality with cases of dispersal, where individuals have left their current group and state and are either re-captured subsequently in a different state, or not re-captured thereafter.

The incorporation of weight and rainfall terms into the multi-state model suggested that a large proportion of the non-breeders that disappeared from groups were individuals that dispersed, rather than individuals that died in situ (Supplementary Table S5). Firstly, heavier individuals were more likely to disappear from their natal group than lighter individuals (MSM: hazard ratio = 1.482, 95% CI [1.298,1.691]), as would be expected if dispersal was more prevalent in older (heavier) individuals. Secondly, individuals were more likely to disappear following periods of increased rainfall (MSM: hazard ratio = 1.355, 95% CI [1.205,1.523]).

Table S5. The effect of body weight and rainfall on the duration of philopatry (timing of dispersal). Transition intensities from a 'multi-state' model fitted to non-breeders first captured at less than one year of age (here only two states considered). Prior to model fitting, the weight term was standardised within each sex, and rainfall was standardised across both sexes. Rainfall represents the total (sum) daily rainfall in the 3 months prior to disappearance/dispersal.

Transition	Intensity	95% CI
in-group non-breeder → in-group non-breeder	-0.530	-0.608, -0.463
in-group non-breeder → dead/disappeared	0.530	0.463, 0.608

Covariate	Hazard ratio	95% CI
Sex Male: in-group non-breeder → dead/disappeared	1.125	0.864, 1.467
Weight: in-group non-breeder → dead/disappeared	1.482	1.298, 1.691
Rainfall: in-group non-breeder → dead/disappeared	1.355	1.205, 1.523

The body condition of single females

To compare the body condition of single females and in-group non-breeding females we performed standardised major axis regressions of body mass on two skeletal size traits: incisor width and body length. We included body length as this is a more conventional linear measurement of skeletal size in mammals. Incisor width was measured as noted in the main text. Body length was measured dorsally from the front of the snout to the tip of the tail using a tape measure (to an accuracy of 1mm). As for incisor width, each measurement was taken in duplicate by two observers, and we here use the average of these two measures. For each skeletal measurement we filtered out any in-group non-breeding females whose skeletal size was less than the minimum size trait found in single females. This meant that the two classes of female were size matched skeletally, such that any difference in condition would reflect increased bodily reserves for a given skeletal size.

Standardised major axis regression was proposed by Peig & Green (2008) as a superior regression method for the scaling of mass on linear body measurements than ordinary least squares methods and has been widely adopted in studies on mammals since. Briefly, we performed a standardised major axis regression of ln body mass against the natural log of each skeletal measurement using the *smatr* package (Warton et al. 2012). In both cases, we initially fitted a model which allowed the slope and intercept to vary according the class of female, but for both skeletal measurement neither the intercept (teeth width LRT: $\chi^2_1 = 1.04$, p = 0.31; body length LRT: $\chi^2_1 = 0.09$, p = 0.76) nor the slope (teeth width LRT: $\chi^2_1 = 1.31$, p = 0.25; body length width LRT: $\chi^2_1 = 2.53$, p = 0.11) significantly different between single females and in-group non-breeders. **This indicates that single females and in-group non-breeders did not differ in their condition**.

We therefore present the results of the scaling relationship where all females were assumed to following the same scaling relationship; modelled by a single slope and intercept (Figure 2 in main text, incisor width intercept = 2.41, 95% CI = -0.08 – 0.56, incisor width slope = 2.57, 95% CI = 2.39 – 2.75; body length intercept = -4.83, 95% CI = -5.33 – -4.33, body length slope = 3.34, 95% CI = 3.17 – 3.52).

The scaled mass index of each measurement can be calculated using the following formula:

$$SMI = M_i \left(\frac{L_0}{L_i}\right)^{b_{SMA}}$$

Where M_i and L_i are the body mass and skeletal measurement of individual *i*, b_{SMA} is the scaling exponent estimated by the SMA regression of *M* on *L*, and L_0 is the arithmetic mean value of the population sample. The scaled mass index of single females and in-group non-breeding females are contrasted below in the inset boxplots.

Within-group recruitment

Experimental pairings

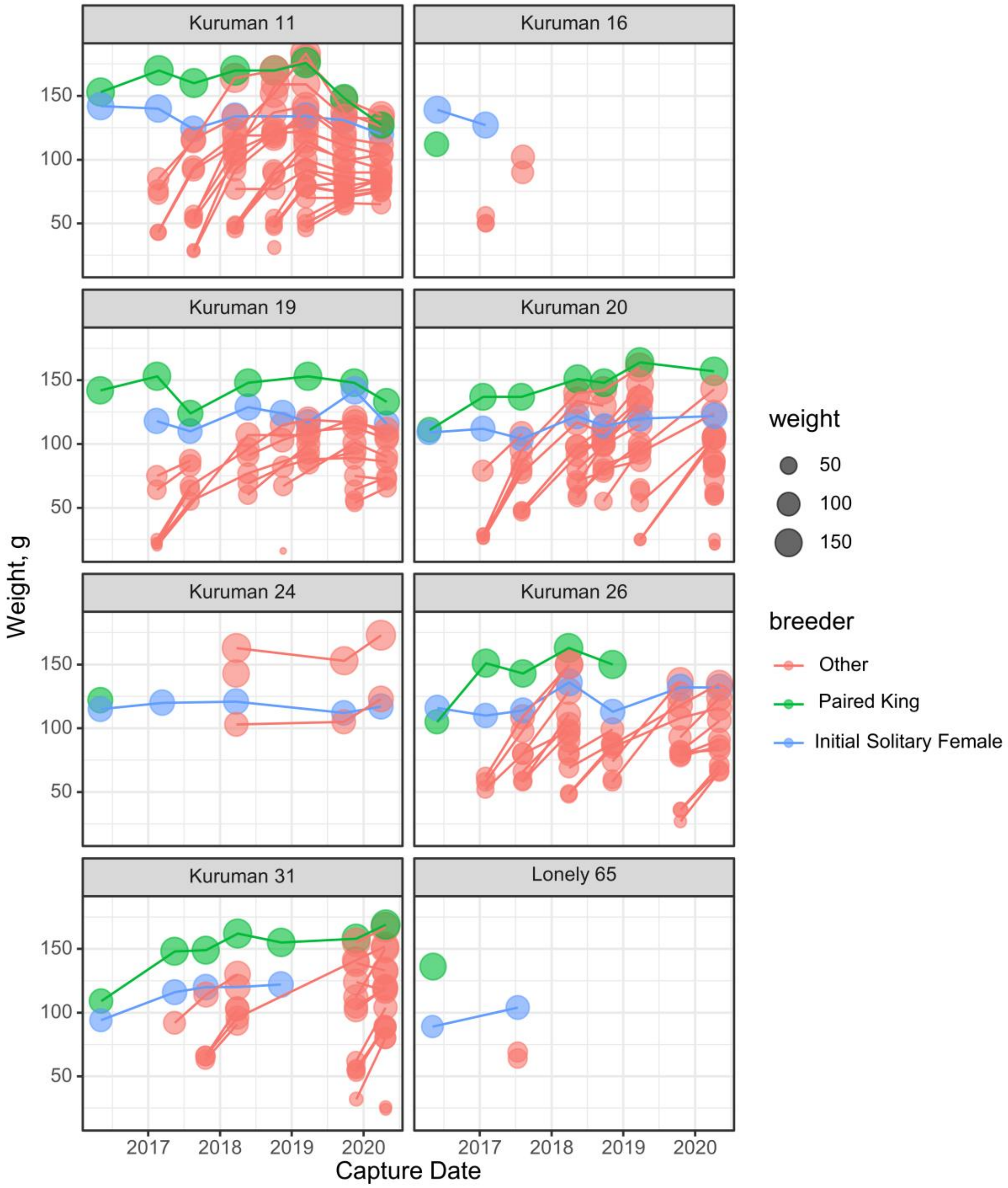

Figure S12. Group structure and trapping history of groups that were created experimentally by the addition of an unfamiliar male to the burrow systems of single females. In most cases newly created pairings commenced breeding shortly after pairing, as indicated by the recruitment of ‘other’ individuals into the group following the initial pairing.

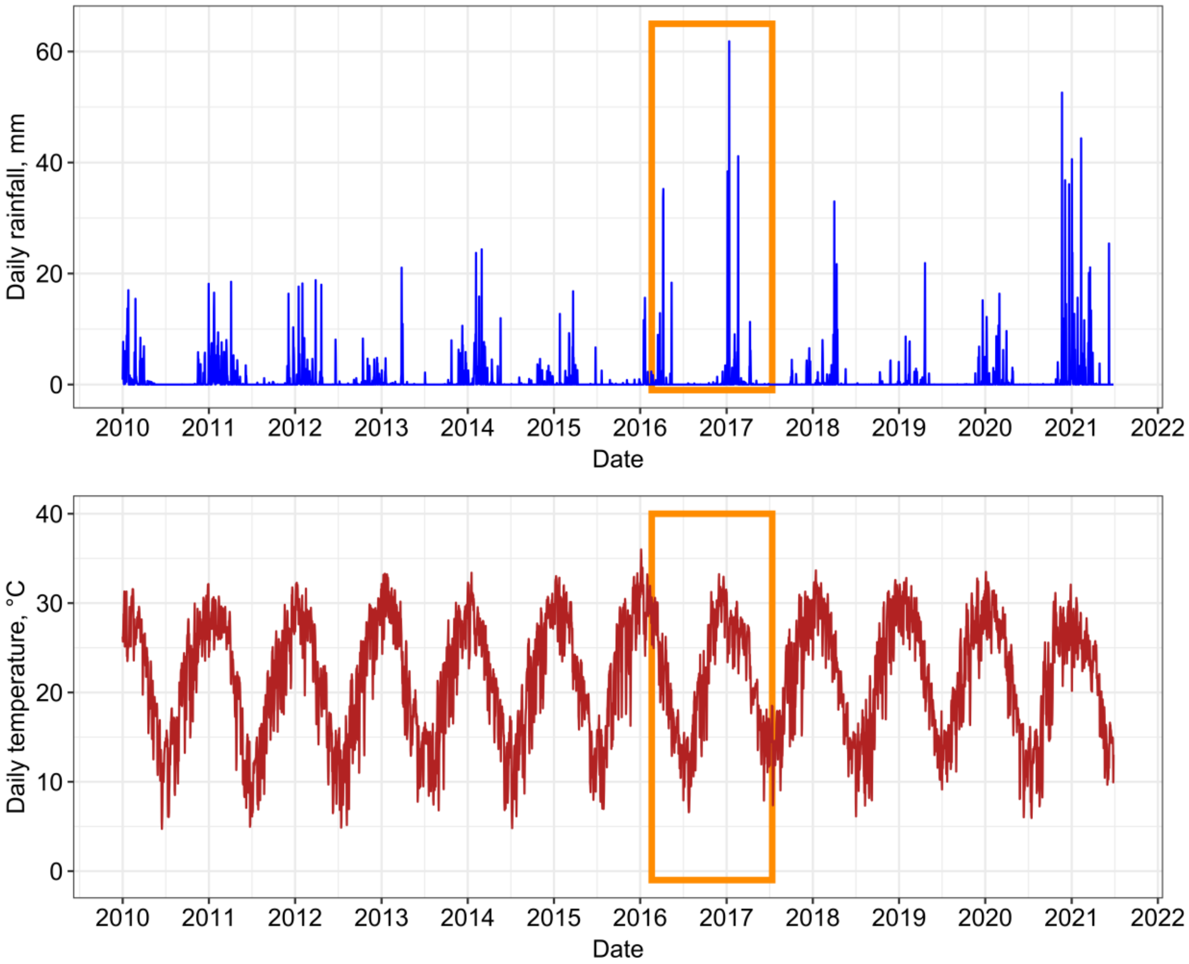

Figure S13. The timing of the experimental pairings in relation to climatic variation at the Kuruman River Reserve. Upper and lower panels document the long-term daily rainfall (mm) and daily average temperature at the field site. The orange rectangle demarcates the period within which experimentally created pairs and established groups (which served as controls) were captured and recaptured. Climate data taken from NASA's GMAO MERRA-2 assimilation model and GEOS 5.12.4 FP-IT.

Table S6. The recruitment rate of offspring into groups in Damaraland mole-rats, longitudinal analysis. Recruitment was modelled with a generalised linear model with negative binomial error distribution. Models were fitted to n = 77 group-level recapture events in 33 distinct breeding groups. All estimates are provided on the link scale (log-link), and continuous variables are z-score transformed. Models also included an offset for the log(time between capture and recapture).

	Mean Estimate	Std. Error.	z-value	p-value
**Fixed effects**				
Intercept	-4.538	0.108		
Group Size	0.216	0.106	2.04	**0.042**
Breeder mass	-0.090	0.089	-1.02	0.309
Rainfall	0.159	0.086	1.85	0.065
**Random effect**	**Variance**	**Std Dev**		
Group ID (n = 33)	0.129	0.359		

Growth and adult body mass

Interval equations

Table S7. Body mass growth. Model summaries provide estimates from the non-linear mixed effects that incorporated group size into von Bertalanffy interval equations. Models were fitted to n = 381 recapture events in females, and n = 456 recapture events in males. Estimates provide the population mean for the fixed effects, the standard deviation for the random effects.

Females	**Mean Estimate**	**Std. Error.**	**t-value, df**	**p-value**
**Fixed effects**				
A	118.26	2.01	58.72, 185	< **0.001**
A_{GS}	-9.42	1.47	6.41, 185	< **0.001**
K	0.00447	0.00003	16.02, 185	< **0.001**
k_{GS}	0.00155	0.00032	4.90, 185	< **0.001**
**Random effect**	**Variance**	**Residual**		
A ~ 1|AnimalID	15.79	9.18		
k ~ 1|AnimalID	Negligible			
Males	**Mean Estimate**	**Std. Error.**	**t-value, df**	**p-value**
**Fixed effects**				
A	149.70	2.84	52.74, 239	< **0.001**
A_{GS}	-8.10	2.02	4.00, 239	< **0.001**
k	0.00368	0.00002	17.90, 239	< **0.001**
k_{GS}	0.00084	0.00020	4.36, 239	**0.001**
**Random effect**	**Variance**	**Residual**		
A ~ 1|AnimalID	21.47	12.51		
k ~ 1|AnimalID	Negligible			

Table S8. Incisor width growth. Model summaries provide estimates from the non-linear mixed effects that incorporated group size into von Bertalanffy interval equations. Models were fitted to n = 328 recapture events in females, and n = 381 recapture events in males. Estimates provide the population mean for the fixed effects, the standard deviation for the random effects.

Females	**Mean Estimate**	**Std. Error.**	**t-value, df**	**p-value**
**Fixed effects**				
A	5.75	0.04	147.60, 145	< **0.001**
A_{GS}	-0.11	0.04	4.17, 145	< **0.001**
K	0.0053	0.0002	26.23, 145	< **0.001**
k_{GS}	0.0031	0.0002	1.37, 145	0.17
**Random effect**	**Variance**	**Residual**		
A ~ 1|AnimalID	0.39	0.16		
k ~ 1|AnimalID	Negligible			
Males	**Mean Estimate**	**Std. Error.**	**t-value, df**	**p-value**
**Fixed effects**				
A	6.48	0.05	132.11, 180	< **0.001**
A_{GS}	-0.05	0.04	1.32, 180	0.19
k	0.00429	0.0001	31.19, 180	< **0.001**
k_{GS}	0.00007	0.0001	0.48, 180	0.64
**Random effect**	**Variance**	**Residual**		
A ~ 1|AnimalID	0.47	0.20		
k ~ 1|AnimalID	Negligible			

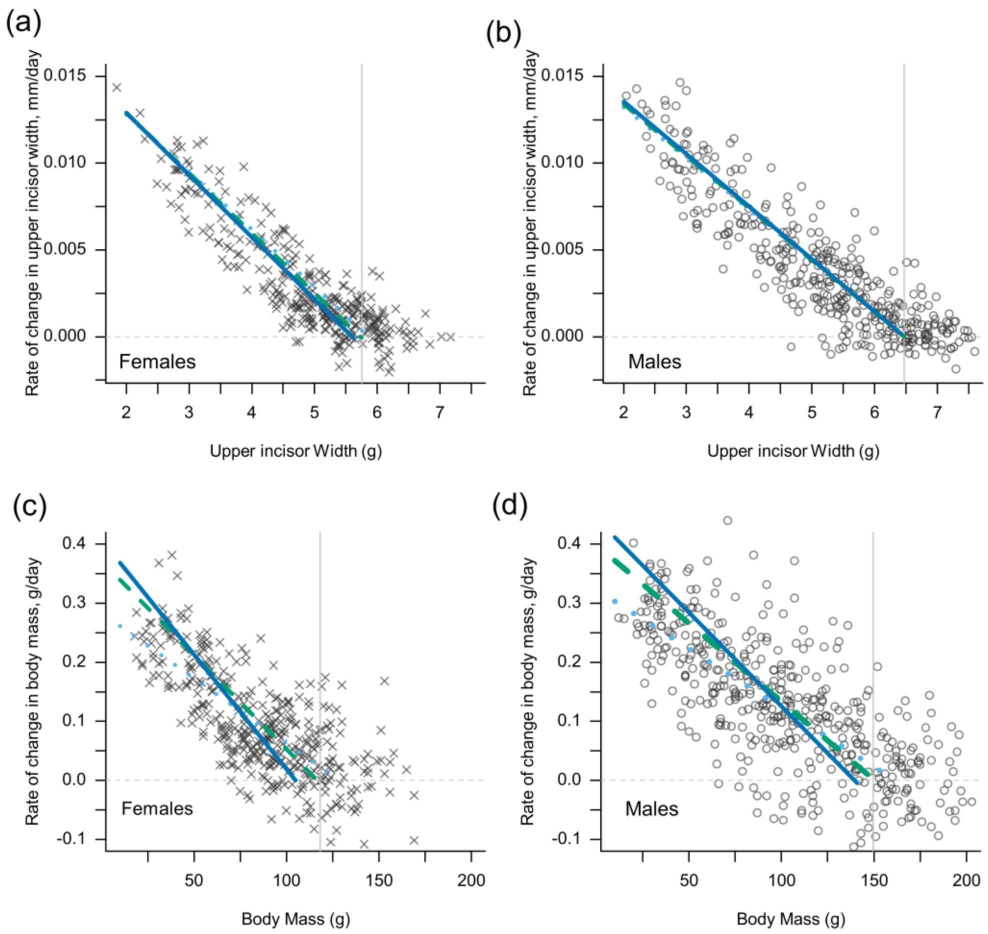

Figure S14.The rate of change in incisor width and body mass for females (a, c) and males (b, d) with variation in initial size and group size. Plots display the data as modelled through interval equations described in the main text. Each point represents the change in incisor width or body mass across two captures - a capture and a recapture. Note that with increasing initial size, the slope of the change in size metric converges on the estimated population-level asymptotes (vertical lines). Small groups (4 individuals, light blue, dotted line), medium groups (12 individuals, turquoise, dashed line), or large groups (20 individuals, blue, solid line).

Group size and adult body mass

Because the parameters of the von-Bertalanffy growth curve covary with one another (as parameterised in the interval equations), it is not possible to dissociate the effect of group size on the growth rate constant, *k*, from the effect of group size on asymptotic mass, *A*. We therefore carried out additional analyses to clarify the effect of group size on adult body mass.

For all the males and females whose growth was modelled by the interval equations (Figure 5, Supplementary Figure S14), we extracted weight information for all the times that they were captured at least one year after first being captured at less than 1 year of age (< 100g for males, < 80g for females). The weights information therefore represented the body mass of all non-breeding individuals captured beyond 1 year of age, with most data coming from individuals far older than this. This generated a data set of 152 weights from 73 males, and 111 weights from 65 females. At the same time, we calculated the average group size that each of these individuals had experienced in their first year of life: for some individuals we had only one value across this period, for others we had up to 3 measures.

The mass of individuals in adulthood of either sex was then modelled according to their current group size and the group size that they experienced in early life (< 1 year of age). The two terms were not strongly correlated (r = 0.26 in males, r = 0.04 in females), and in both male and female data sets the variance inflation factor of the two terms was < 1.07. Both early life group size and adult group size could therefore be fitted together in the same model. Adult body mass was fitted in a linear mixed effects model (Gaussian error), with average group size in the first year specified as a continuous variable, and group size in adulthood specified as a categorical variable with three levels: small, medium, and large, separated into the tertiles in each data set. For males at 1-9 (small), 10-16 (medium), >16 (large); for females at 1-7 (small), 8-15 (medium), >15 (large). An additional variable noting the quarterly period of the year was also included (Jan-Mar; Apr-Jun; Jul-Sep; Oct-De). Group size in early life was standardised prior to model fitting, and a random effect of individual identity, nested within group identity, was specified in each case. Models were fitted in the *lme4* package in R. Model outputs are presented in Supplementary Table S9 and visualised in Supplementary Figure S15.

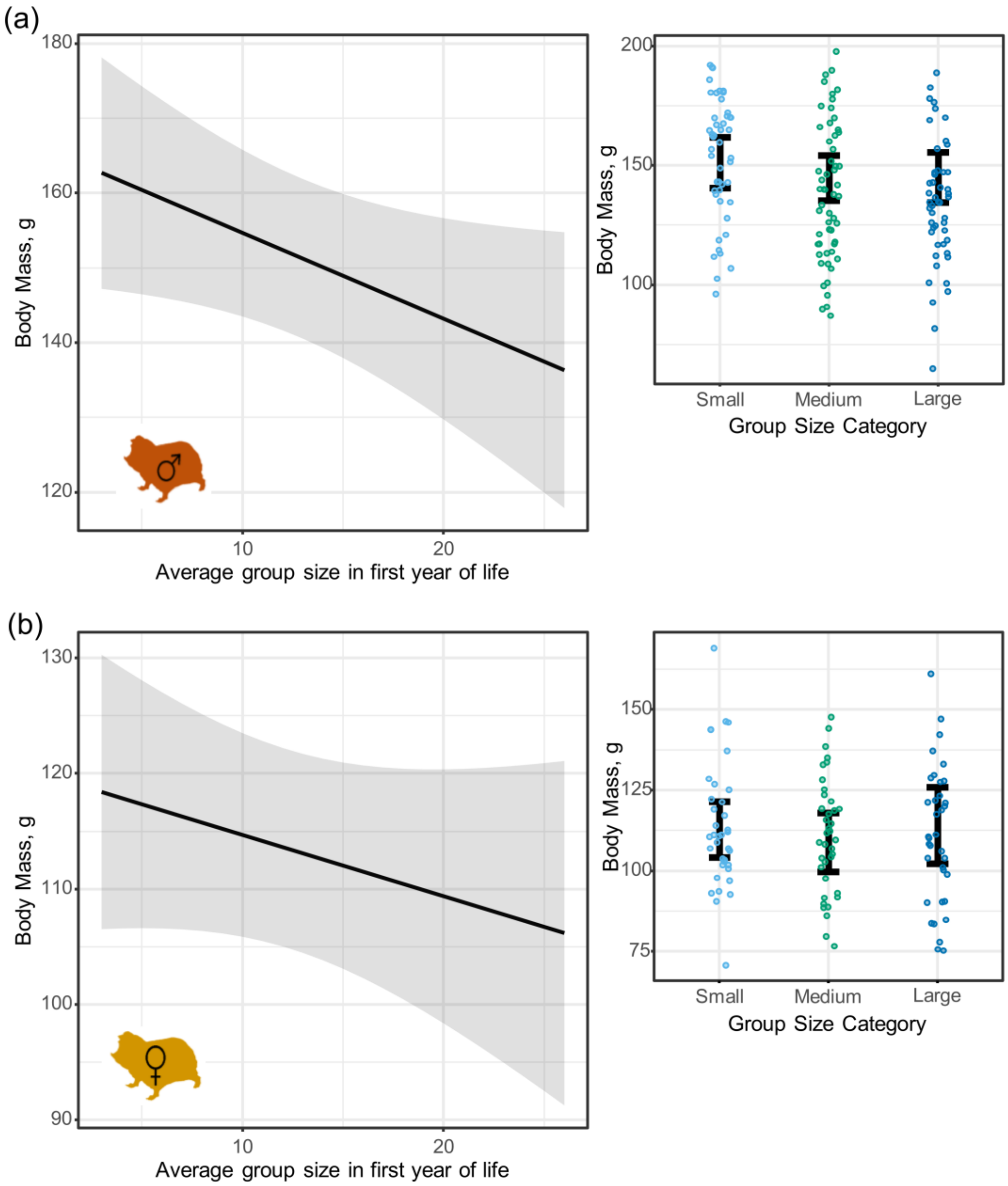

Figure S15. Adult body mass of non-breeding male (a) and female (b) Damaraland mole-rats. Left panels display the predicted effect of average group size in the first year of life on adult mass (< 100g for males, < 80g for females), as estimated from linear mixed effects models (Table S9). In both cases, increases in group size during early life reduces body mass in adulthood. By contrast, current group size in adulthood has no effect on adult body mass, as displayed in the right panels. Points denote the raw data across all individuals, with error bars denoting the predicted marginal effects across three group size categories. Group size categories were separated at the tertiles within each data set; for males at 1-9 (small), 10-16 (medium), >16 (large); for females at 1-7 (small), 8-15 (medium), >15 (large). Post-hoc tests performed on the linear mixed effects models found that in neither sex did group size in adulthood have a significant effect on adult body mass ($p > 0.46$ in all contrasts). Note that group size in adulthood was categorised largely for the purpose of visualisation; treating the term as a continuous variable did not qualitatively affect the results.

Table S9. The adult body mass of mole-rat non-breeders. Female and male mass was modelled using a linear mixed effects model with Gaussian error distribution. In either case, models were fitted to non-breeding individuals that were captured at least one year after they were first captured at less than 1 year of age (< 100g for males, < 80g for females). They therefore represent the average body mass of all non-breeding individuals captured beyond 1 year of age, with most data coming from individuals far older than this. Group size categories were separated at the tertiles within each data set; for males at 1-9 (small), 10-16 (medium), >16 (large); for females at 1-7 (small), 8-15 (medium), >15 (large). n = 65 unique females from 25 groups. n = 73 unique males from 23 groups. See Supplementary Figure S14 for plotted predictions.

Females (n = 111)	**Mean Estimate**	**Std. Error.**	**t-value, df**	**Chisq, df**	**p-value**
**Fixed effects**					
Intercept	112.95	4.41			
Adult group size: medium	-4.12	4.62	-0.892	1.23, 2	0.54
Adult group size: large	1.15	6.27	0.183		
Yearly quarter: second	-4.55	3.87	-1.178	1.90, 3	0.59
Yearly quarter: third	-4.15	4.11	-1.009		
Yearly quarter: fourth	-2.06	3.97	-0.511		
Early life group size	3.08	2.62	-1.176	1.23, 1	0.27
**Random effect**	**Variance**	**Std Dev**			
Animal ID:Group ID	162.2	12.73			
Animal ID	114.6	10.71			
Residual	128.5	11.34			
Males (n = 152)	**Mean Estimate**	**Std. Error.**	**t-value, df**		
**Fixed effects**					
Intercept	151.30	5.44			
Adult group size: medium	-6.39	4.49	-1.423	2.13, 2	0.34
Adult group size: large	-6.15	5.36	-1.148		
Yearly quarter: second	-14.22	3.36	-4.238	27.15, 3	<0.001
Yearly quarter: third	-15.53	3.24	4.789		
Yearly quarter: fourth	-12.03	3.57	-3.373		
Early life group size	-6.56	3.33	-1.967	2.99, 1	0.084
**Random effect**	**Variance**	**Std Dev**			
Animal ID:Group ID	493.2	22.21			
Animal ID	159.4	12.62			
Residual	141.0	11.88			

Analyses summary

Table S10. Summary of the analyses carried out in the paper, with information on the data used.

Analysis	Data	Total rows of data	Total unique inds	Total unique groups	Included incomplete captures	Method	Comments
Status-related survivorship in females	All capture events from females, irrespective of age (weight) at first capture. Additional rows generated for when individuals disappeared or were right-censored.<br><br>“*Fates_Allfemales.csv*”	F: 1315 (953 captures)	F: 362	80	Yes	Multi-state Markov model.	- Right-censored individuals that were still present for the last trapping window at either study location.<br>- Four states: i- *in-group non-breeding female*, ii- *single female*, iii- *breeding female*, iv - *disappeared/dead*<br>- Figure 3. Table S4.
Natal dispersal	“*FieldMR_Femalephilopatry.csv*”<br>“*FieldMR_Malephilopatry.csv*”	1571 (1375 captures)	F: 227<br>M: 272	56	Yes	Two-state Markov model	- Right-censored individuals that were still present for the last trapping window at either study location.<br>- Two states: i- *in-group non-breeder*, ii- *disappeared/dead/known disperser*<br>- Figure S11. Table S5.
Growth: body mass	Body mass across successive capture events (a capture and recapture)<br><br>“*FieldMR_Growth_BodyMass.csv*”	F: 381<br>M: 456	F: 193<br>M: 214	F: 48<br>Male: 39	NA	Non-linear mixed effects model with von Bertalanffy interval equation.<br>Males and females analysed separately.	- Removed breeding females so that pregnancy weights were excluded.<br>- Only chose recaptures that occurred 100-365 days after the first capture.<br>- Figure 5, Figure S14. Table S7<br>- Follow-up analysis to check the effect of group size on asymptotic mass (above; Figure S14, Table S9).
Growth: incisor width	Incisor width across successive capture events (a capture and recapture)<br><br>“*FieldMR_Growth_TeethWidth.csv*”	F: 328<br>M: 381	F: 180<br>M: 198	F: 47<br>Male: 38	NA	Non-linear mixed effects model with von Bertalanffy interval equation.<br>Males and females analysed separately.	- Only chose recaptures that occurred 100-365 days after the first capture.<br>- Figure S14. Table S8.

Body condition of females	All body mass measurements with an associated skeletal trait measurement (either teeth width of body length)<br><br>"*FieldMR_SingleFemaleCondition_BodyLength.csv*"<br>"*FieldMR_SingleFemaleCondition_TeethWidth.csv*"	F incisor width: 353<br><br>F body length: 419	F incisor width: 177<br><br>F body length: 213	F incisor width: 64<br><br>F body length: 67	NA	Scaled major axis regression of ln(body mass) ~ ln(skeletal trait).<br><br>In both cases, a separate slope of intercept for single females was not supported.	-Included information from females that were either an in-group non-breeder or a single female. As the main question was whether single females had a similar body condition to in-group non-breeding females, we only considered in-group non-breeders when their skeletal trait was greater than the smallest recorded value in single females (i.e. removing young females still in their natal group to ensure this didn't bias allometric scaling).<br>- Figure 2.
Within-group recruitment rate (longitudinal)	All complete group captures and recaptures separated by 100-365 days.<br><br>"*FieldMR_Recruitment_Longitudinal.csv*"	78 capture-recapture events	NA	33 groups	No	Generalised linear mixed effects model with Poisson error distribution.<br>Included offset term for time interval.	-Within-group recruits classed as all new individuals in the group on recapture that were less than year of age (body mass < 80g for females, body mass < 100g for males")"<br>- Only included groups if capture history indicated the presence of a breeding female.<br>- Figure 4. Table S6.
Within-group recruitment rate (experimental)	The number of new recruits on the first recapture of experimentally created pairs, which were compared to the number of recruits in established groups captured across the same time period.<br><br>"*FieldMR_Recruitment_Experimental.csv*"	Pairings: 8<br><br>Time-matched groups: 9	NA	NA	No	t-test on recruitment rate	- Figure 4.

Supporting Information References
(excluding those already noted below Table S2)

Beaudette, D,. Skovlin, J., Rocker, S., Brown, A. (2022) *soilDB*: Soil database interface. R Package v 2.7.5.

Bennett, N.C., & Faulkes, C.G. (2021) Damaraland and naked mole-rats: convergence of social evolution. In Walter D. Koenig & J. L. Dickinson (Eds.), *Cooperative Breeding in Vertebrates* (pp. 338–352).

Burland, T.M., Bennett, N.C., Jarvis, J.U.M. (2002) Eusociality in African mole-rats: new insights from patterns of genetic relatedness in the Damaraland mole-rat (*Cryptomys damarensis*). *Proc. Roy. Soc. B.,* 269: 1025-1030.

Burland, T.M., Bennett, N.C., Jarvis, J.U.M., Faulkes, C.G. (2004) Colony structure and parentage in wild colonies of cooperatively breeding Damaraland mole-rats suggest incest avoidance alone may not maintain reproductive skew. Molecular Ecology, 13: 2371-2379.

Faulkes, C.G., Bennett, N.C., Bruford, M.W., O'Brien, H.P., Aguilar, G.H., & Jarvis, J.U.M. (1997) Ecological constraints drive social evolution in the African mole-rats. *Proc. Roy. Soc. B.,* 264: 1619-1627.

Finn, K.T., Parker, D.M., Bennett, N.C., Zöttl, M. (2018) Contrasts in body size and growth sugges that high population density results in faster pace of life in Damaraland mole-rats (*Fukomys damarensis*). *Canadian Journal of Zoology*, 96: 920-927.

Hengl, T., Mendes de Jesus, J., Heuvelink, G.B.M., Ruiperez Gonzalez, M., Kilibarda, M., Blagotić, A., et al. (2017) SoilGrids250m: Global gridded soil information based on machine learning. *PLoS ONE*, 12(2): e0169748.

Jarvis, J.U.M., Bennett, N.C., Spinks,, A.C. (1998) Food availability and foraging by wild colonies of Damaraland mole-rats (*Cryptomys damarensis*): implications for sociality. *Oecologia* 113: 290-298.

Lovegrove, B., Knight-Eloff, A. (1988). Soil and burrow temperatures, and the resource characteristics of the social mole-rat *Cryptomys damarensis* (Bathyergidae) in the Kalahari Desert. *J. Zoology*, 216: 403-416.

Lövy, M., Šklíba, J., Burda, H., Chitaukali, W. N., Šumbera, R. (2012) Ecological characteristics in habitats of two African mole-rat species with different social systems in an area of sympatry: implications for the mole-rat social evolution. *J. Zoology*, 286: 145-153.

Mynhardt , S., Harris-Barnes, L., Bloomer, P., Bennett, N.C. (2021) Spatial population genetic structure and colony dynamics in Damaraland mole-rats (*Fukomys damarensis*) from the southern Kalahari. *BMC Ecology and Evolution,* 21: 221.

Patzenhauerová, H., Šklíba, J., Bryja, J., Šumbera, R. (2013). Parental analysis of Ansell's mole-rat family groups indicates a high reproductive skew despite relatively relaxed ecological constraints on dispersal. Mol. Biol. 22, 4988–5000.

Peig, J., & Green, A.J. (2009) New perspectives for estimating body condition from mass/length data: the scaled mass index as an alternative method. *Oikos*, 118: 1883-1891.

Spinks, A., Bennett, N.C. Jarvis, J.U.M. (2000) A comparison of the ecology of two populations of the common mole-rat, *Cryptomys hottentotus hottentotus*: the effect of aridity on food, foraging and body mass. *Oecologia*, 125: 341-349.

Torrents Ticó, M., Bennett, N. C., Jarvis, J. U. M., & Zöttl, M. (2018). Sex-differences in timing and context of dispersal in Damaraland mole-rats (*Fukomys damarensis*). *J. Zoology*, 306: 252-257.

Warton, D.I., Duursma, R.A., Falster, D.S., & Taskinen, S. (2012) smatr 3- an R package for estimation of allometric lines. *Methods in Ecology & Evolution,* 3: 257-259.

Young, A.J., & Bennett, N.C. (2010) Morphological divergence of breeders and helpers in wild Damaraland mole-rat societies. *Evolution*, 64, 3190-3197.

Young, A. J., Jarvis, J. U. M., Barnaville, J., & Bennett, N. C. (2015). Workforce Effects and the Evolution of Complex Sociality in Wild Damaraland Mole Rats. *The American Naturalist*, 186(2): 302-311.
